# Unbound Brain-to-Plasma Partition Coefficient, K_p,uu,brain_—a Game Changing Parameter for CNS Drug Discovery and Development

**DOI:** 10.1007/s11095-022-03246-6

**Published:** 2022-04-11

**Authors:** Irena Loryan, Andreas Reichel, Bo Feng, Christoffer Bundgaard, Christopher Shaffer, Cory Kalvass, Dallas Bednarczyk, Denise Morrison, Dominique Lesuisse, Edmund Hoppe, Georg C. Terstappen, Holger Fischer, Li Di, Nicola Colclough, Scott Summerfield, Stephen T. Buckley, Tristan S. Maurer, Markus Fridén

**Affiliations:** 1 Department of Pharmacy, Uppsala University, Box 580, Uppsala, Sweden; 2 DMPK M&S, Pharma R&D, Bayer AG, Berlin, Germany; 3 DMPK, Vertex Pharmaceuticals, Boston, Massachusetts 02210 USA; 4 Translational DMPK, H. Lundbeck A/S, Copenhagen, Denmark; 5 External Innovation, Research & Development, Biogen Inc., Cambridge, Massachusetts USA; 6 DMPK-BA, AbbVie, Inc., North Chicago, Illinois USA; 7 Pharmacokinetic Sciences, Novartis Institutes for BioMedical Research, Cambridge, Massachusetts USA; 8 DMPK, Janssen Research & Development, Beerse, Belgium; 9 Rare and Neurological Diseases, Sanofi, Chilly Mazarin, France; 10 DMPK, Boehringer-Ingelheim Pharma GmbH & Co. KG, Biberach, Germany; 11 Cambrian Biopharma, New York, New York USA; 12 Translational PK/PD and Clinical Pharmacology, Pharmaceutical Sciences, Roche Pharma Research & Early Development, Roche Innovation Center Basel, Basel, Switzerland; 13 Pharmacokinetics, Dynamics and Metabolism, Pfizer Worldwide Research and Development, Groton, Connecticut USA; 14 Oncology DMPK, Oncology R&D, AstraZeneca, Cambridge, UK; 15 Bioanalysis Immunogenicity and Biomarkers, GSK, Gunnels Wood Road, Stevenage, SG1 2NY Hertfordshire UK; 16 Global Research Technologies, Novo Nordisk A/S, Måløv, Denmark; 17 Pharmacokinetics, Dynamics and Metabolism, Pfizer Worldwide Research and Development, Cambridge, Massachusetts USA; 18 Inhalation Product Development, Pharmaceutical Technology & Development, Operations, AstraZeneca, Gothenburg, Sweden

**Keywords:** blood–brain barrier, CNS drug development, drug transport, neuropharmacokinetics, unbound brain-to-plasma partition coefficient, K_p,uu,brain_

## Abstract

**Purpose:**

More than 15 years have passed since the first description of the unbound brain-to-plasma partition coefficient (K_p,uu,brain_) by Prof. Margareta Hammarlund-Udenaes, which was enabled by advancements in experimental methodologies including cerebral microdialysis. Since then, growing knowledge and data continue to support the notion that the unbound (free) concentration of a drug at the site of action, such as the brain, is the driving force for pharmacological responses. Towards this end, K_p,uu,brain_ is the key parameter to obtain unbound brain concentrations from unbound plasma concentrations.

**Methods:**

To understand the importance and impact of the K_p,uu,brain_ concept in contemporary drug discovery and development, a survey has been conducted amongst major pharmaceutical companies based in Europe and the USA. Here, we present the results from this survey which consisted of 47 questions addressing: 1) Background information of the companies, 2) Implementation, 3) Application areas, 4) Methodology, 5) Impact and 6) Future perspectives.

**Results and conclusions:**

From the responses, it is clear that the majority of the companies (93%) has established a common understanding across disciplines of the concept and utility of K_p,uu,brain_ as compared to other parameters related to brain exposure. Adoption of the K_p,uu,brain_ concept has been mainly driven by individual scientists advocating its application in the various companies rather than by a top-down approach. Remarkably, 79% of all responders describe the portfolio impact of K_p,uu,brain_ implementation in their companies as ‘game-changing’. Although most companies (74%) consider the current toolbox for K_p,uu,brain_ assessment and its validation satisfactory for drug discovery and early development, areas of improvement and future research to better understand human brain pharmacokinetics/pharmacodynamics translation have been identified.

**Supplementary Information:**

The online version contains supplementary material available at 10.1007/s11095-022-03246-6.

## Introduction

At the time of writing, more than 15 years have passed since the first presentation of the unbound brain-to-plasma drug partition coefficient (K_p,uu,brain_) to the research community by Gupta *et al*. in 2006 (1). By analogy to the partition coefficients used previously, i.e. total brain-to-plasma drug partition coefficient (K_p_) and total brain-to-unbound plasma partition coefficient (K_p,u_), the authors denoted this novel unbound partition coefficient, K_p,uu_ (later for clarity called K_p,uu,brain_). It exclusively describes the unbound drug concentration in the brain relative to blood at equilibrium and is determined only by the net influx and efflux clearances, CL_in_ and CL_out_, and not by any subsequent partitioning into brain cells (2). As it was proposed by Hammarlund-Udenaes *et al*., “K_p,uu,brain_ gives a direct quantitative description of how the blood–brain barrier (BBB) handles the drug regarding passive transport and active influx/efflux” (2). K_p,uu,brain_ can be assessed by using the area under the curve (AUC) of unbound drug concentration – time profile in brain and plasma after single dosing. Alternatively, the steady-state unbound concentrations of drug in brain interstitial fluid (ISF, C_u,brain,ss_) and in plasma (C_u,plasma,ss_) can be used (Eq. 1).

1 $${K}_{p,uu,brain}=\frac{{CL}_{ in}}{{CL}_{out}}=\frac{AU{C}_{ u,brain}}{{AUC}_{u,plasma}}=\frac{{C}_{u,brain,ss}}{{C}_{u,plasma,ss}}$$

The introduction of K_p,uu,brain_ as a measure of the extent of BBB transport of primarily small molecular weight drugs has been facilitated by advancements in intra-cerebral microdialysis techniques (3–12) and accumulating evidence that neither total nor unbound plasma concentration of a drug can be directly used with high confidence as a surrogate for the brain ISF concentration in many cases (1, 13–22). The latter reinforces the need to measure the unbound brain drug concentration. It is important to note that, so far, direct assessment of unbound drug concentrations in the brain and plasma is still only possible with the microdialysis sampling technique and, hence, it is considered as a gold reference standard for validation of novel methods where unbound tissue drug concentrations are in focus. Yet, due to the inherent features of microdialysis and the multiple challenges (and costs) associated with its implementation and performance, this type of validation is unfortunately rarely conducted (13, 23–26).

**Fig. 1:**
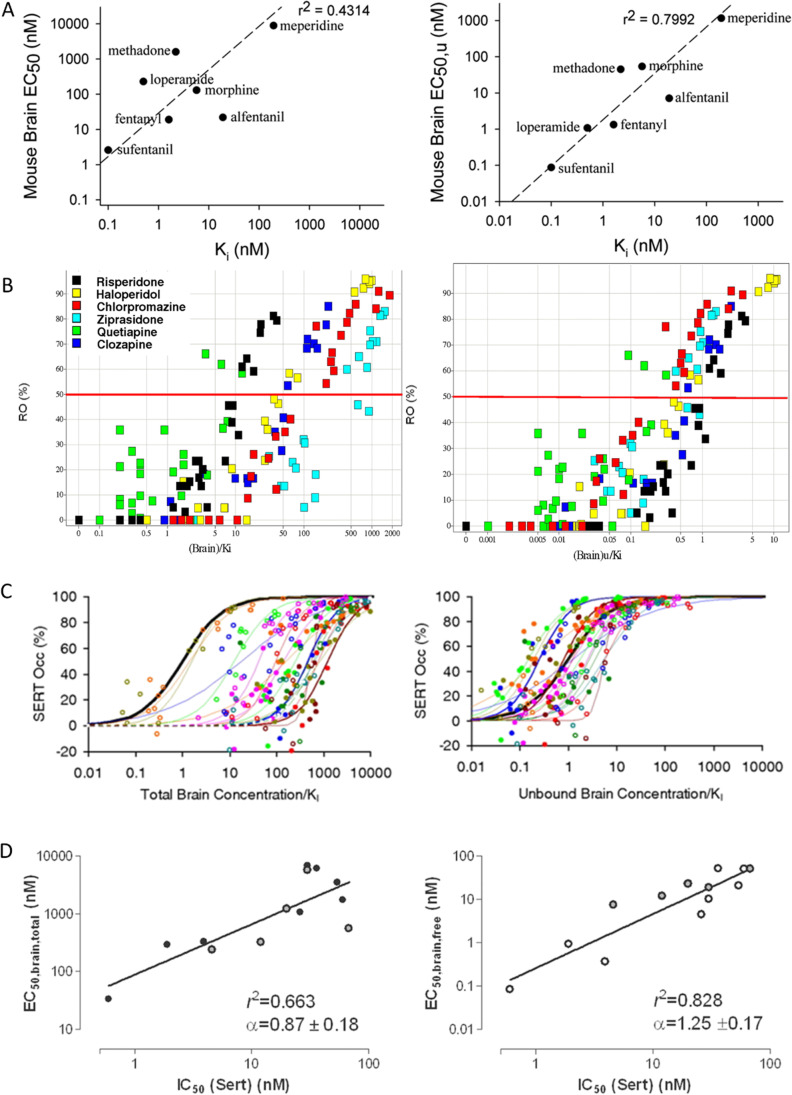
Collated experimental evidence of estimated unbound brain drug concentration (*right panel*) better correlating with effect than measured total brain concentration (*left panel*) across series of molecules. (**A**) Correlation between *in vitro* potency (Ki) and brain EC50 for antinociception in mice (47). (**B**) Correlation between *in vitro* potency (Ki) normalized brain concentration and occupancy of dopamine 2 (D_2_) receptor in the brain (44). (**C**) Correlation between *in vitro* potency (Ki) normalized brain concentration and occupancy of serotonin transporter (SERT) in the brain (43). (**D**) Correlation between *in vitro* potency (Ki) and brain EC50 of serotonin transporter (Sert) occupancy (49). Figures reproduced with permission from the respective publishers.

In addition to the advancements in methodological aspects, the adoption of the K_p,uu,brain_ concept was facilitated by substantial improvements in the fundamental understanding of transport of non-electrolytes across the membrane in the light of the Free Drug Hypothesis (8, 11, 13, 27–38). The latter has been well summarized by Smith *et al*. with the distinction between two critical parts of the hypothesis (37). Part I: at steady state, the unbound drug concentration is the same on both sides of any biomembrane, with several exclusions from the rule, i.e., cases including involvement of efflux and influx transporters as it often occurs at the blood–brain interface emphasizing the requirement on K_p,uu,brain_ estimation. Part II: the free drug concentration at the site of action, the therapeutic target biophase, is the species that exerts pharmacological activity. Regarding the second postulate, knowledge has also accumulated to support the long-held presumption that the unbound concentration of drug in the brain is the driving force for pharmacological response (15, 22, 39–49) (Fig. 1).

In parallel, a better understanding of drug-tissue binding processes has led to the development of *in vitro* methods including equilibrium dialysis with one of the first applications of this technique being the assessment of fraction of unbound drug in plasma, f_u,plasma_ (27, 50–55). Since the implementation of the equilibrium dialysis technique, countless studies have been performed based on the assumption that *in vivo*-measured f_u,plasma_ is comparable to the *in vitro*-measured value, however, the validity of such equivalency was first directly established using the microdialysis technique by Dubey *et al*. (13) and later confirmed in several studies (14, 41, 56).

Investigation of drug binding in brain tissue homogenate using equilibrium dialysis has also been broadly tested to estimate the fraction of unbound drug in the brain, f_u,brain_ (35, 54, 57, 58). Proposition of a high-throughput equilibrium dialysis method (for simplicity often called brain homogenate method) by Kalvass and Maurer (40) made the assessment of unbound drug concentration in the brain based on measured total brain drug concentrations possible and also applicable for use in industrial settings (41–44, 47, 59–65). Currently, this method is accepted as a standard approach in the pharmaceutical industry. However, the methodological limitations inherent to homogenizing the tissue provided impetus for developing the organotypic brain slice assay for investigation of both drug binding and cellular uptake into the brain tissue (24, 66, 67). Development of a brain slice assay suitable for the evaluation of the unbound volume of drug distribution in the brain, V_u,brain,_ for test compounds in a drug discovery setting, which was validated against data generated by *in vivo* brain microdialysis, represented a significant advancement in the field (68, 69). Comparison of f_u,brain_ and V_u,brain_ values, which are inversely correlated to each other (f_u,brain_≈1/V_u,brain_), showed that the brain slice assay was advantageous for investigation of weak bases/acids as well as compounds with active transport across the cell plasma membrane (70–72). On the basis that the brain slice assay represented overall tissue drug uptake and the brain homogenate method essentially represented intracellular binding, it was proposed that an unbound partition coefficient of the cell, K_p,uu,cell_ could be calculated to represent intracellular exposure to unbound drug (24).

Substantial progress in the ability to obtain reliable measures of the extent of drug-tissue binding both in plasma and brain facilitated the paradigm shift from K_p,brain_ to K_p,uu,brain_ in an industrial setting. Currently, K_p,uu,brain_ is often assessed using Eq. 2 with multiple examples of such implementation for both the brain as an overall estimate and in specific brain regions of interest (25, 40–43, 47, 59, 61, 72–80).

2 $${K}_{p,uu,brain}\approx \frac{{K}_{p,brain}}{{f}_{u,plasma}*{V}_{u,brain}}\approx \frac{{K}_{p,brain}}{{f}_{u,plasma}*\frac{1}{{f}_{u,brain}}}\approx {K}_{p,brain}*\frac{{f}_{u,brain}}{{f}_{u,plasma}}$$

The K_p,uu,brain_ concept has also been accepted by brain positron emission tomography (PET) imaging experts and with use examples described not limited to just rodent species (77, 81–87). In addition, the approach has been used in combination with mass spectrometry imaging, which improved the spatial resolution of the method allowing investigation of K_p,uu,brain_ in small brain regions and subregions (88).

Generation of larger rodent K_p,uu,brain_ datasets using Eq. 2 facilitated the development of quantitative structure–activity relationship (QSAR) in silico models (25, 89–94). Furthermore and in parallel, the striving to minimize animal usage and increase throughput led to the development of various *in vitro* cell culture BBB models (95–102). For instance, values of apparent permeability (P_app_) in cell monolayers have been used to estimate the time required to reach distribution equilibrium between brain and plasma, and bidirectional transporter assays of P-glycoprotein (P-gp) and breast cancer resistant protein (BCRP) can provide useful insights on efflux transport at the BBB (59, 96, 98, 103–111). The utility of cell culture models to predict the rate and the extent of BBB drug transport *in vivo* has been widely applied in the pharmaceutical industry. In addition, mathematical modeling including physiologically-based pharmacokinetic (PBPK) models with focus on unbound CNS drug concentrations are currently widening the perspectives and usage of the K_p,uu,brain_ concept (112–116). Whilst this introduction above provides a high-level summary of the development of K_p,uu,brain_ concepts and methodology, there are little real world ‘data’ available on how these are implemented across pharmaceutical industry and used today in contemporary drug discovery. To that end, a survey was designed and completed by the authors of this manuscript representing major pharmaceutical companies involved in drug discovery and development in Europe and the USA. Key findings from the survey are summarized in this paper. The results indicate that the K_p,uu,brain_ concept has been adopted broadly throughout the pharmaceutical industry to enable effective design of CNS therapeutics and minimize central side-effects.

## Materials and Methods

The perspectives and practices described in this paper regarding industrial implementation of K_p,uu,brain_ concept and methodology were captured by the authors with the aim to represent the current status and contemporary views of the pharmaceutical industry. The group of industry-affiliated authors was brought together on the basis of their publication record in peer-reviewed scientific journals and presentations at scientific conferences, aiming to have representatives from major pharmaceutical companies. It was recognized upfront that we might not be able to ensure complete representation from pharmaceutical companies, e.g., beyond a certain size or level of involvement of small molecule CNS drug research. Almost all invited contributors chose to participate and co-author the paper. To initiate the discussion and facilitate the capture of perspectives and practices, the assembled group agreed to construct and conduct a survey to probe relevant areas related to the K_p,uu,brain_ concept and methodology implementation. All authors contributed and agreed to the final survey questions, and for the results to be handled and published anonymously through an Uppsala University internal survey platform (KURT; https://doit.medfarm.uu.se/bin/kurt3/?lang=en). As only one response per question was collected from each company, all authors were asked to represent their company in the best possible way by engaging with relevant company functions and individual experts where appropriate before responding. The majority of authors described themselves as belonging to the discipline of ‘drug metabolism and pharmacokinetics’ (DMPK), which is typically the disciplines responsible for K_p,uu,brain_ measurements and interpretations. Other departments such as medicinal chemistry, neurology and biosciences were also represented in the survey.

The survey included single/multiple choice, yes/no and free-text components, and consisted of 47 questions aimed at gathering information in six different areas with regards to K_p,uu,brain_: 1. Background information of author’s companies, 2. Implementation, 3. Application areas, 4. Methodology, 5. Impact and 6. Future perspectives (see supplemental material, S1, questionnaire). All responses were collected in the period of September 21, 2021 to October 14, 2021 (see supplemental material, S2, KURT autogenerated summary of the results). Data analysis was conducted using Microsoft Excel (Microsoft Corporation, USA).

## Results and Discussion

### Responders are Involved in the Development of a Wide Range of Modalities Across Diverse Therapeutic Areas

A total of 14 responses to the survey were obtained capturing broad representation from pharmaceutical companies involved in drug discovery and development in Europe and the USA. Of the 14 respondents, 13 (93%) were from large pharmaceutical companies (with > 5,000 employees) and only one (7%) from a mid-size pharmaceutical company (Supplemental Material, S2). Responders were primarily affiliated with the department of DMPK, but may have also incorporated views and input from other disciplines or departments such as medicinal chemistry, neurology or biosciences. The top three therapeutic areas that the participating companies are working in are neuroscience, oncology, and inflammation. Many companies also are working on metabolic, cardiovascular and infectious diseases. Consistent with industry trends, surveyed companies were engaged in both small molecule and biologics research. It is recognized in this context that use of K_p,uu,brain_ concept and methodology is broader than the companies that are represented in the author list and the individuals that have completed the survey.

### Bottom-up-Driven Transformation of an Entrenched Paradigm

**Fig. 2:**
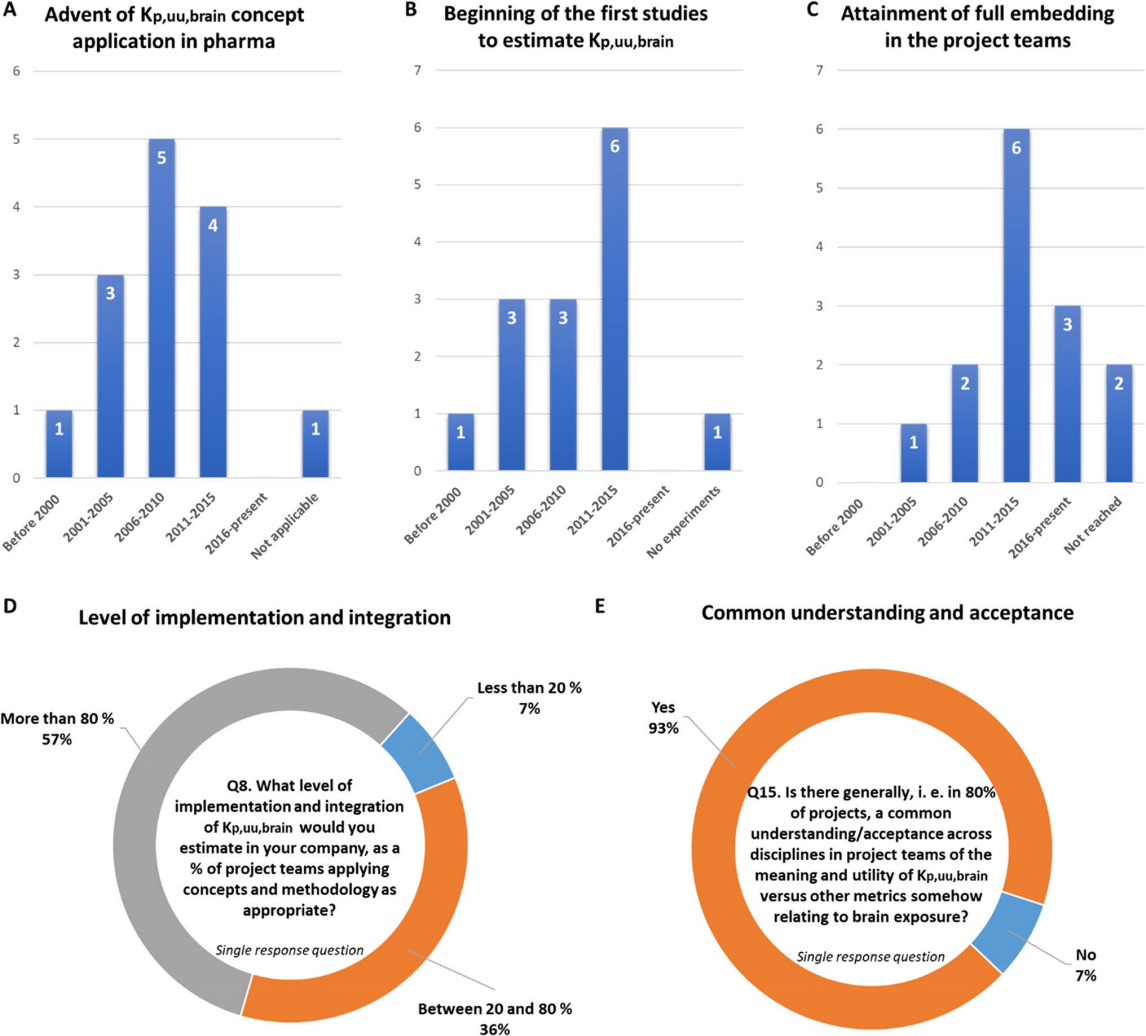
Summary on a timeline of implementation (**A-C**) and integration (**C**, **D**) of K_p,uu,brain_ concept in pharmaceutical companies. NB: The Fig. 2 is based on the following questions: Q6 (A), Q7(B), and Q9 (C).

Implementation and integration of the K_p,uu,brain_ concept in the pharmaceutical industry was explored in the ‘Implementation’ section of the survey (Questions (Q) 6–16, Supplemental material, S1, S2) to understand aspects of timing, key drivers, mechanisms of implementation and current status. Remarkably, prior to 2000 (7%) and in the period of 2001–2005 (21%), project teams or key scientists had already begun to advocate the concept of the K_p,uu,brain_ (Fig. 2A). This was sparked by accumulating evidence supporting the need to measure unbound drug concentration in the blood as well as in the brain (8, 11–17, 23, 26, 36, 40, 41, 102, 104, 117–122). The peak for internal understanding and endorsement of the importance of K_p,uu,brain_ was 2006–2010 (35%). This was also the time period that several key research papers emerged, which largely shaped the K_p,uu,brain_ concept as it is today (1, 2, 21, 22, 24, 25, 37, 42–48, 59, 61, 62, 68, 123–131). Project teams found the K_p,uu,brain_ impactful, as evidenced by the percent of companies that started to measure K_p,uu,brain_ by 2010 (50%, Fig. 2B) and fully embedded the approach by 2015 (64%, Fig. 2C). Despite sporadic recognition of the importance of K_p,uu,brain_ prior to 2005, 63% of companies had fully embedded the concept into project teams by 2015 (Fig. 2C).

Currently, 57% of responding companies indicated that the level of implementation and integration of the K_p,uu,brain_ concept is more than 80% (Fig. 2D). In addition, it is clear that the majority of the companies (93%) have been successful in achieving a common understanding/acceptance across multiple disciplines of the meaning and utility of K_p,uu,brain_ as compared to other parameters/measurements related to brain exposure (Fig. 2E). Taken together, the K_p,uu,brain_ concept seems to be well-embedded within project teams as an important criterion to understand unbound drug distribution in the brain, such that teams are not misled by parameters calculated solely from total concentrations. K_p,uu,brain_ is arguably one of the most important parameters to be optimised by medicinal chemistry design in the context of therapies for CNS diseases to maximize brain exposure or for peripheral targets to minimize CNS toxicity.

Based on the survey, the main drivers for introducing and implementing the K_p,uu,brain_ concept into the pharmaceutical companies were: 1) a general shift in paradigm and increased scientific rigour in pharmacology and PK (37%), 2) difficulties to explain what are the PK drivers for efficacy (33%) and 3) unexpected and unexplained CNS side effects (23%). It is noteworthy that the key mechanism of implementation of the K_p,uu,brain_ concept (S1, Q11) was not following a top-down approach, but rather driven by individual scientists advocating the application of K_p,uu,brain_ concepts in project teams (28%) and provision of data to a number of selected projects as examples to prove its usefulness (56%). It might be speculated that these observations more broadly reflect the process of paradigm change in pharmaceutical industry where diversity of opinions and engagement are increasingly embraced in the interest of fostering innovation. It also highlights the importance of leading changes by individuals at the grass-root level.

The survey results show that about half of the companies (49%) have not adopted a default company-wide strategy for determination of K_p,uu,brain_ as part of the lead optimization/compound screening schemes (S1, Q 12). It is more driven on the basis of specific project team decision-making tailored towards the needs of individual drug discovery programs (12/14 answers). This suggests that a “one-sized fits all” approach is not well suited to the fast-paced and ever-changing environment of drug discovery. This may also indicate a level of sophistication in how project teams function where it is in appropriate to have guidance that is overly prescriptive. K_p,uu,brain_ in conjunction with the desired target product profile is one of the many parameters (e.g., potency, PK or pharmacodynamic (PD), safety) that need to be optimized. Hence, a holistic assessment of all data is required to determine whether a given compound should be progressed to the next stage. In fact, it may be possible to advance a compound without an “ideal” K_p,uu,brain_ value, if sufficient target engagement is predicted to be achievable within its safety profile.

Across all companies, CNS exposure assessment is considered a core responsibility of the DMPK departments (S2, Q13). Interestingly, only 28% of the companies were running the respective experiments entirely in-house (S2, Q14). Most companies use CRO for routine K_p,uu,brain_ and related parameters measurements. However, data interpretation and integration of K_p,uu,brain_ remain a function of DMPK experts.

### K_p,uu,brain_ as a Parameter with a Direct Quantitative Link to the Estimate of Therapeutic Dose

Applications of the K_p,uu,brain_ concept (S1, S2, Q17) were broad in the pharmaceutical industry. Neuropharmacokinetic (neuroPK) profiling from an efficacy standpoint are widely applied (12/14, Q17), where K_p,uu,brain_ is used as selection criteria for entry into resource-intensive *in vivo* pharmacology studies. Furthermore, almost all responders (13/14) stated that K_p,uu,brain_ was used to define PK/PD relationships for CNS effects and/or prediction of therapeutic dose. As an example, one responder described a general approach where project teams apply the unbound brain concentrations derived from predicted plasma concentrations and a K_p,uu,brain_ experiment to assess theoretical target coverage (e.g., unbound brain concentration, C_u,brain_ vs. *in vitro* target-derived the half maximal inhibitory (IC_50_) /efficacy concentration (EC_50_)). Hence, a compound with a moderate K_p,uu,brain_ (e.g., 0.2) may still be considered for progression if it is both potent and displaying otherwise favourable predicted human PK (provided acceptable peripheral side effects and therapeutic index). K_p,uu,brain_ is not only used for rank order of compounds to guide medicinal chemistry design, but also applied to predict brain unbound drug exposure and dose. The majority of the responders (11/14, Q20) indicated that the numerical value of K_p,uu,brain_ are used directly to predict therapeutic dose. Additionally, the survey results suggest that other important information can be derived from the experimental data of a K_p,uu,brain_ study, such as *in vitro-in vivo* potency assessment, PK/PD and safety. This is a unique aspect as the unbound drug concentrations can be used in project teams across disciplines, and not just be as a ‘stand-alone’ K_p,uu,brain_ data point. In essence, the work conducted around the K_p,uu,brain_ concept has supported the evolution of the free drug hypothesis in CNS drug research and paved the way towards using unbound drug concentrations as drivers of efficacy.

Another strong theme from the survey outcome is the impact of determining *in vivo* K_p,uu,brain_ values in enabling validation of higher throughput in vitro assays and developing *in vitro – in vivo* extrapolations with good predictability. Remarkably, all responders (14/14, Q17) indicated that by successfully correlating measured K_p,uu,brain_ with *in vitro* assays (e.g., P-gp and BCRP efflux ratio), it has become possible to use these *in vitro* methods to efficiently and reliably screen, identify and prioritize compounds with desired K_p,uu,brain_. The latter is striking as 72% of the responders use transporter-transfected cell lines (e.g., MDR1 and BCRP-Madin-Darby Canine Kidney cells) rather than brain endothelial cell culture systems for testing compounds (Fig. 4F), supporting the notion that these non-BBB cell systems are robust and highly effective screening tools for efflux transporter activity that can be translated to* in vivo*.

**Fig. 3:**
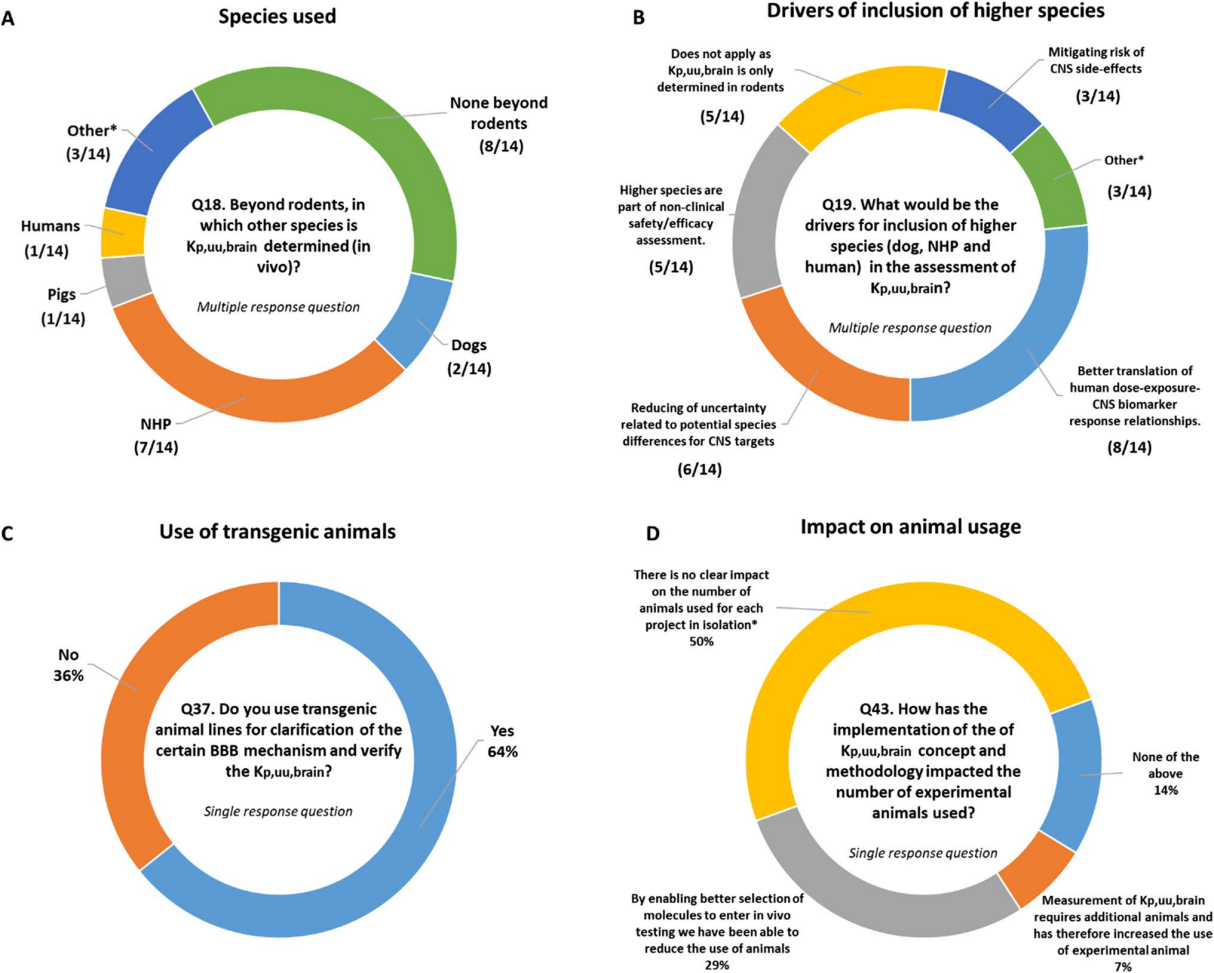
Overview on the usage of mammals for assessment of K_p,uu,brain_ *in vivo* (**A**), justification of a need to include higher species (**B**), usage of transgenic animals (**C**) as well as overall impact of implementation of K_p,uu,brain_ methodology on the usage of preclinical animals (**D**). NB: In A Other*- Non-rodents only at late discovery stage (close to candidate selection) to strengthen human translation; There may occasionally be PET data used to calculate K_p,uu_ in non-human primates (NHP) and humans; NHP PET receptor occupancy data along with other data is used to infer NHP K_p,uu,brain_, but K_p,uu,brain_ has not been measured directly from NHP. In B Other*—Mitigation of species dependent transporter mediated efflux (e.g., BCRP in monkey), K_p,uu,brain_ in other species is being rationally approached rather than experimentally determined, typically applying all relevant contextual data, NHP PET receptor occupancy data along with other data is used to infer NHP K_p,uu,brain_, but K_p,uu,brain_ has not been measured directly from NHP. In D *—There is no clear impact on the number of animals used for each project in isolation. However, by enabling better selection of compounds it has increased the probability of success and can therefore be seen as a reduction in animal use. It is critical to mention that all procedures performed on animals were in accordance with regulations and established guidelines and were reviewed and approved by Institutional Animal Care and Use Committee.

As drug discovery programs move towards candidate selection and beyond, companies (7/14) may occasionally include higher species (e.g., non-human primates (NHP)), in brain exposure studies to probe specific questions. The key drivers for inclusion of higher (non-rodent) species (Fig. 3B) were better translation of human dose-exposure-CNS biomarker-response relationships and reducing uncertainty related to potential species differences in K_p,uu,brain_ for projects with CNS targets.

Other areas of application include the evaluation of CNS off-target (10/14) and CNS on-target (11/14) safety assessment. A small number of respondents (3/14, Q21) indicated that K_p,uu,brain_ is used for assessment of the effects of disease and age on transports at the BBB. Several examples were provided where these questions had been addressed. For example, K_p,uu,brain_ was determined in specific pharmacological animal models that may influence BBB integrity (e.g., drug induced seizure animal model or transgenic mouse models for neurological applications like Alzheimer’s disease), or for a target with known age-dependent expression and pharmacological effect in mice. In addition, more than half of the responders (64%) have utilized transgenic animals to investigate certain BBB transport mechanisms or to verify K_p,uu,brain_ values from other studies (Fig. 3C). Most responders (93%, Q22) did not perform any investigation of transporter drug-drug interactions at the BBB. This is because clinical modulation of efflux transport by P-gp and BCRP at the human BBB is unlikely as supported by the international transporter consortium evidence-based position paper (133).

Finally, 86% of responders (12/14, Q23) typically consider the brain interstitial fluid drug concentration to adequately represent exposure in brain cells. Two companies mentioned that additional studies were performed to determine K_p,uu,cell_ in order to obtain intracellular drug concentration. Rightly or wrongly, it seems to be a common assumption that unbound brain drug concentration in interstitial fluid, C_u,ISF,_ is a reasonable surrogate for unbound intracellular drug concentration, C_u,ICF_. Future research would be important to pressure-test this assumption further.

### Broad Consensus Around Key Methodological Aspects of K_p,uu,brain_ Determination

**Fig. 4:**
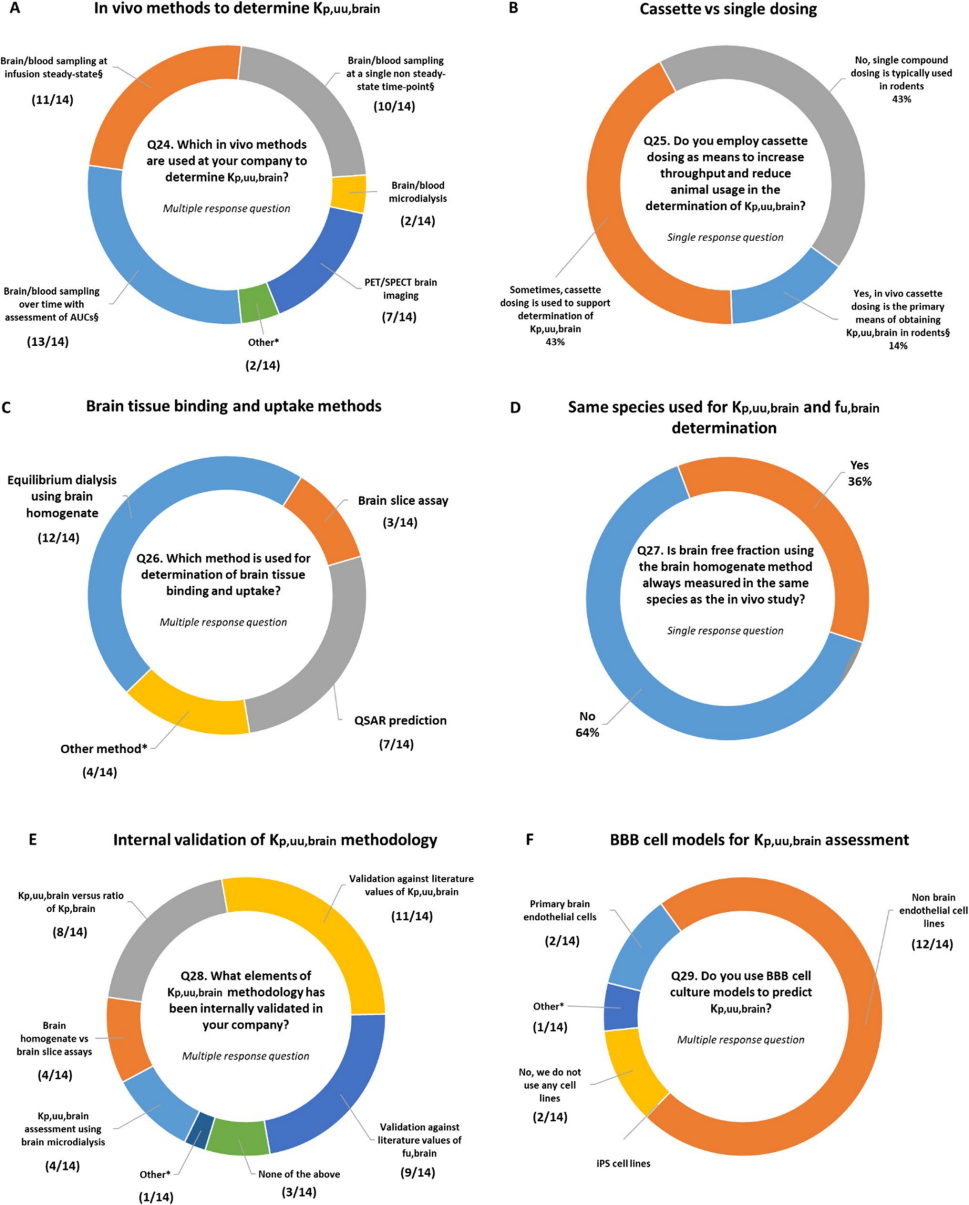
Summary on methodologies used for the assessment of K_p,uu,brain_ and brain tissue binding (**A**, **B, C, D**), status on the internal validation of the key methodologies (**E**) and the usage of the blood–brain barrier cell culture models by pharmaceutical companies. NB: In A §—in preclinical animals followed by binding correction in respective tissue; Other*—Plans to do PET imaging for compounds in Development, e.g., for compounds targeting brain tumors, Not yet broadly implemented. In B §—Determination from animals dosed with single compound is typically limited to PD experiments, higher species studies or other exceptions. In C Other method*—Muscle to brain ratio to estimate efflux at the BBB (132); LIMBA—Lipid membrane binding assay (87); Imaging; Ultracentrifugation and ultrafiltration. In E – Other*—validation in characterizing cross-compound and cross-series relationship between unbound brain drug concentration and pharmacodynamic responses measured preclinically. In F Other*—No absolute prediction of K_p,uu_ performed from cell studies; rather qualitative information around *in vitro*—*in vivo* efflux correlations.

Technical aspects and practices of K_p,uu,brain_ determination using *in vivo*, *in vitro* and in silico approaches are covered by Q24-40 (S1, S2). Starting with the *in vivo* experimental setups, it was evident that the majority of companies employ plasma and brain sampling to determine K_p,brain_ and correct for binding in plasma and brain to obtain K_p,uu,brain_. Only two companies indicated the use of brain microdialysis, which is often considered as the gold standard in K_p,uu,brain_ determination. This is most likely a consequence of technical challenges (along with costs) to establish this technique and efficiently screen large numbers of compounds. The most widespread experimental setup for determining K_p,brain_ assesses AUCs in brain and plasma (13/14 responders) followed by single time point determinations using steady-state infusion (11/14) and non-steady state conditions (10/14) (Fig. 4A). In addition, brain imaging including PET is also used by 50% of responders. In terms of usage of single vs. cassette dosing in K_p,uu,brain_ determination (Fig. 4B), the survey results show a split with 43% of the companies typically using single compound dosing; while 43% sometimes practice cassette dosing. Cassette dosing has been investigated previously with rather encouraging results supporting this approach for increasing the throughput of compound testing (74, 134, 135). Two companies use *in vivo* cassette dosing as the primary means of obtaining K_p,uu,brain_ in rodents.

Questions 32–34 (S1, S2) explore practical options of how and when to conduct K_p,uu,brain_ determination in relation to other ongoing *in vivo* activities with the same molecule. About a third of the companies routinely measure brain and plasma samples in standard PK and PD studies (Q32). The majority of companies (70%) obtain brain exposure in PD studies from plasma exposure and K_p,uu,brain_ determined in a dedicated study. In case of a need to determine the temporal aspect of CNS exposure in pharmacology (e.g., unbound brain concentration at specific time-points such as pre-dose trough levels in repeat-dose studies), 56% of companies would conduct sampling of brain to enable calculation of exposure at these time points, whereas only 35% would use the plasma concentration and K_p,uu,brain_ determined from a separate dedicated study or the same study. For the determination of brain tissue binding and uptake required for estimation of unbound brain drug concentration based on measured total concentration, 12/14 companies (Q26, Fig. 4C) utilize high-throughput equilibrium dialysis with the brain homogenate method (40, 41). A more labour-intensive brain slice assay (24, 67–69) is used by only 3 companies. Interestingly, 7/14 companies developed and implemented QSAR predictions for brain tissue binding and uptake. In addition, some utilize other less common approaches, such as a novel assay called LIMBA (lipid membrane binding assay) utilizing porcine brain polar lipids (87), ultracentrifugation and ultrafiltration. When estimating the fraction of unbound drug in the brain using the brain homogenate method, 64% of the companies typically use a single species, e.g., rat, and assume species-independent drug brain tissue binding properties (Fig. 4D), echoing the key findings on the lack of interspecies differences in brain homogenates (59, 63).

As might have been anticipated, most companies (11/14) had internally validated at least some element of K_p,uu,brain_ methodology in comparison with published literature values with a predominant focus on K_p,uu,brain_ (27%) and f_u,brain_ (22%) (Fig. 4E). Only 4 out of 14 responders performed validation of K_p,uu,brain_ via cerebral microdialysis, which again points to the technical challenges (including costs) of setting up and conducting large numbers of studies employing this method. Validation by characterizing cross-compound and cross-series relationships between unbound brain drug concentration and PD responses measured preclinically has been performed in one company. Interestingly, only 4 companies have investigated the brain slice assays in relation to the brain homogenate method.

Companies generally put emphasis on screening compounds for efflux transport using *in vitro* models (e.g., transfected MDCK, LLC-PK1 cells), most commonly employing bi-directional transport studies (97, 98, 106, 107, 136, 137) and more recently unidirectional transport with or without transporter inhibitors (138). The impact of efflux transporters on brain exposure and K_p,uu,brain_ has been well documented (98, 100, 105–108, 136, 137, 139). K_p,uu,brain_ models have been developed using efflux ratios of P-gp and BCRP in multiple species, and some are comparing to human values of K_p,uu,CSF_ or K_p,uu, brain_ derived from PD effect.

More than half of the companies (56%, Q36) have established values for K_p,uu,brain_ which are considered ‘high’ or ‘low’ with rather broad consensus on threshold for high K_p,uu,brain_ > 0.3 to 0.5. In general, a compound with K_p,uu,brain_ > 0.3—0.5 is accounted as brain penetrant considering experimental variability (42). This approach could be considered highly effective in providing guidance to medicinal chemists in the design of molecules with improved characteristics of brain penetration. However, whether a given unbound drug concentration in the brain is sufficient or not to elicit pharmacological activities is dependent on a number of additional factors that require more comprehensive PK/PD modelling. Nevertheless, K_p,uu,brain_ cut-off values offer an initial calibration of the brain penetration potential of compounds. In light of the complexity of the question, it is notable that some companies consider an acceptable K_p,uu,brain_ to be any value that allows a desired therapeutic index and dosing regimen.

From the methodological perspective, similar to standard drug discovery PK studies, most companies (77%, Q38) often do not apply any predefined acceptance criteria, such as number of replicates, positive/negative controls or assessment of uncertainly propagated into the composite K_p,uu,brain_ estimate. This is consistent with the common practices in that positive and negative controls are not typically used in *in vivo* PK or neuroPK studies in drug discovery. Once PK procedures and technical details have been established with validation compounds, no additional controls are added for studying new compounds. This is because different animals, formulations, and doses are used for new compounds. It is difficult to obtain a true control. As such, animals within the group are served as a control for assay variability. Studies will be repeated if variability is too high or abnormal data are observed. Typically, two to three animals are used for neuroPK studies. Some companies (21%) developed acceptance criteria regarding inter-individual variability in K_p,uu,brain_ determined across three rats. In this context, several companies have outlined that a better understanding of where the variability comes from in the assessment of K_p,uu,brain_ is a key question needing further attention (Q47).

Following the trends in predictive sciences, 8 out of 14 companies (Q30) have developed and use QSAR models derived by machine learning algorithms, PBPK and other means to predict K_p,uu,brain_ from the chemical structure and/or physicochemical properties. Some responders have developed individual in-house in silico models for f_u,brain_, f_u,plasma_, P-gp and BCRP efflux ratios, and passive permeability although these separate models have not been combined together and validated for the ability to predict K_p,uu,brain_. The in silico P-gp and BCRP efflux ratios have be used as an input parameter to predict K_p,uu,brain_ (106, 137). PBPK modelling for brain tissue with input parameters derived from physicochemical properties and/or *in vitro* data is a growing field with six companies employing this approach. The development of mechanistic mathematical models (e.g., PBPK) is acknowledged as an area requiring further research to improve the prediction accuracy (Fig. 6B). Wide applications of QSAR and other in silico models in predicting brain K_p,uu,brain_, f_u,brain_, f_u,plasma_, and efflux by P-gp/BCRP are great advancements in the field. They are powerful tools to enable medicinal chemistry design to enhance or restrict brain penetration prior to synthesis. These approaches significantly reduce resources needed for *in vitro* and *in vivo* assays, cycle times and animal usage. As higher quality data become available for structurally diverse compounds, the predictability will continue to improve. In fact, establishment of truly predictable QSAR models has been recognized as an important topic for development in the next 15 years (Fig. 6C). High quality transporter proteomics data as an addition to already existing knowledge (140–143) also play a tremendous role in further refining in silico models in predicting *in vivo* brain K_p,uu,brain_.

The often-asked question on the appropriateness of CSF as a surrogate of unbound concentration of a compound in brain interstitial fluid deserves further clarifications (25, 124, 144–149). Kalvass *et al*. (133) discussed that the matter comes down to whether CSF represents the brain interstitium for moderate-to-high permeable compounds without major efflux much better than for compounds with extensive efflux. The former is of questionable practical value, since for such molecules the unbound plasma concentration would be considered adequate to represent unbound brain concentration. The above is also reflected in the survey results. Collection of CSF in rodents is performed occasionally by 57% of responders (Q31), and only 14% apply it as a common practice. Some companies use CSF sampling mainly for large molecules (e.g., monoclonal antibodies) or for compounds for which no known transporters are involved in transport across CNS barriers. It is important to mention that further understanding of CSF exposure, also in relation to its sampling site, is considered one of the critical aspects needed for successful translation from preclinical species to patients (Fig. 6B).

**Fig. 5:**
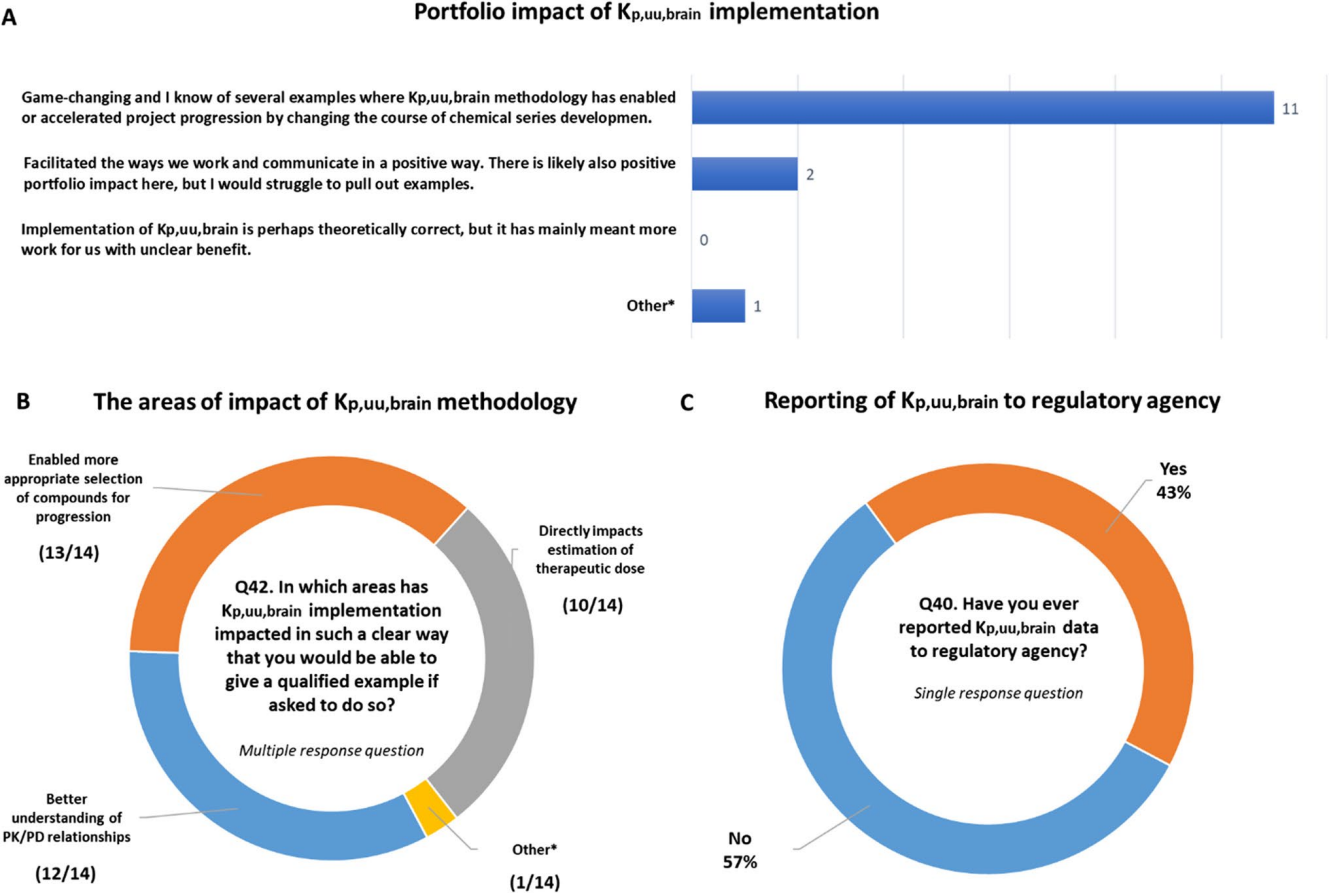
Overview of portfolio impact of K_p,uu,brain_ implementation (**A**) with outline of the key areas of impact (**B**) as well as the practice of reporting K_p,uu,brain_ to regulatory agencies (**C**). NB: In A (Q41) Other*- We haves struggled with its implementation and are still at stage where its implementation is still in its infancy. In B Other*—Not yet sufficient examples.

Another indirect method for evaluation of BBB-penetration is quantitative whole-body autoradiography (QWBA). To understand the general perception of QWBA for assessment of BBB penetration in the pharmaceutical companies, the question was posed as to how such data are interpreted and reported for submission to regulatory authorities (Q39). QWBA data were generally judged as qualitative data mainly with the potential in cases of low/no intensity to suggest poor brain penetration. Many companies do not use QWBA data for K_p,uu,brain_ assessment nor BBB penetration, because data represent total drug-related radioactivity including parent molecule plus potential metabolites. K_p,uu,brain_ values have been included in the submission documents to regulatory agencies with 57% of the responders (Q40) having already done so (Fig. 5C). This inclusion of K_p,uu,brain_ in regulatory submission documents is in relation to PK/PD modelling, pharmacological effects and toxicological evaluation as part of the filing process of drug candidates included in Investigator's Brochure, Investigational New Drug Applications and other documents.

### Game-Changing Impact in Drug Discovery and Development

There is strong and consistent testimony in support of the positive impact of the K_p,uu,brain_ concept on drug discovery and development portfolios. Most companies (11/14, response to Q 41 (S1, S2)) recognize that the implementation of K_p,uu,brain_ in drug discovery was ‘game-changing’. Responders were able to provide several examples where K_p,uu,brain_ methodology has enabled or accelerated project progression by changing the course of chemical series development, or enabled critical understanding of CNS PK/PD. Details and specifics of this impact was explored further within the group of authors and it became clear that there is a spectrum of impact areas and positive outcomes in drug discovery and development that are linked to K_p,uu,brain_ implementation.

Delivering on the early promises of the methodology, several authors recognised that K_p,uu,brain_ has enabled more appropriate selection of compounds for progression. The weight of impact seems strongest in shaping an efficient workflow in drug discovery putting in place an efficient ‘screening cascade’ or ‘design-make-test-analyse cycle’ wherein *in vitro* methodology such as efflux ratios in P-gp and BCRP transfected cell lines are used for high throughput compound profiling. This development would not have been possible without the correlation to relevant *in vivo* data that have been provided by K_p,uu,brain_ methodology. Furthermore, with confidence in the *in vitro – in vivo* correlation, it is possible to take the next steps by considering the results from in silico QSAR models, thus, further impacting the molecular design process prior to synthesis. These approaches already significantly reduce the resources needed for *in vitro* and *in vivo* assays including animal usage and shorter cycle times.

The establishment of the resource efficient process described above has required not only technological development of *in vivo*, *in vitro* and in silico methodology, but also the creation of a commonly accepted and understood PK/PD framework. This framework integrates results from quantitative assays measuring drug potency, efflux transport, metabolic clearance, etc. In the context of a broader understanding of target engagement requirements for efficacy through a holistic assessment of the molecule’s potential of becoming a drug – culminating in the ‘predicted therapeutic human dose’. This PK/PD based framework and specifically the free drug hypothesis, is unquestionably implemented in both CNS and non-CNS drug discovery of major pharma companies. It is the view of the authors that the early developments of K_p,uu,brain_ concepts and methods for the brain, having to address one of the most complex organs in the body from a drug exposure point of view, has effectively served to evolve the free drug hypothesis in CNS drug research, and paved the way towards using the unbound concentrations as driver of PD in quantitative modelling of PK/PD relationships. Interestingly, several of the authors responded that K_p,uu,brain_ concepts and methodology are being applied to other organs and tissues such as liver, lung, muscle, heart, adipose, nerve or even cells of organs (79, 150–156).

As evident from the discussion above, the role of K_p,uu,brain_ goes far beyond categorically labelling drug molecules as being brain penetrant or non-brain penetrant, extending into areas of predicting clinical efficacy and safety. The responses to Q42 (Fig. 5A) showed that a majority of the companies had seen examples of portfolio impact in areas of 1) better understanding PK/PD relationships, 2) better selection of compounds, and 3) quantitative input to the prediction of human dose. An illustrative example of impact across all these areas is presented as a case example from AstraZeneca describing the development AZD1390, a brain penetrant inhibitor of ataxia-telangiectasia mutated (ATM) serine/threonine protein kinase for the treatment of glioblastoma (Box 1).


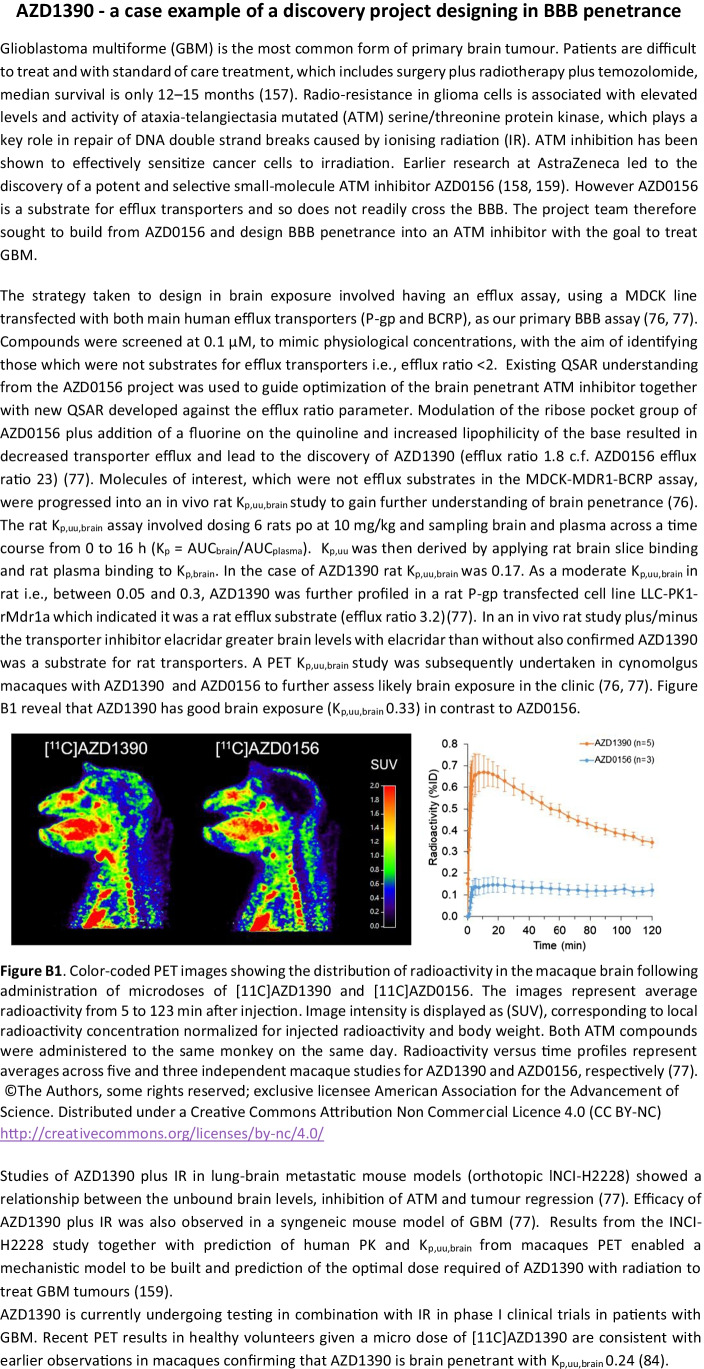


During the preparation of this manuscript, it became known that pharmaceutical companies are now beginning to include K_p,uu,brain_ data in regulatory submissions, which in consideration of the inertia of the regulatory landscape, testifies to the impact K_p,uu,brain_ is having. The emerging inclusion of K_p,uu,brain_ data in regulatory submissions likely reflects how the parameter is linked to the assessment of CNS safety in early clinical trials where regulators increasingly request the details underpinning the predicted therapeutic dose and MABEL, i.e., Minimal Anticipated Biological Effect Level (161).

## Future Perspectives

**Fig. 6:**
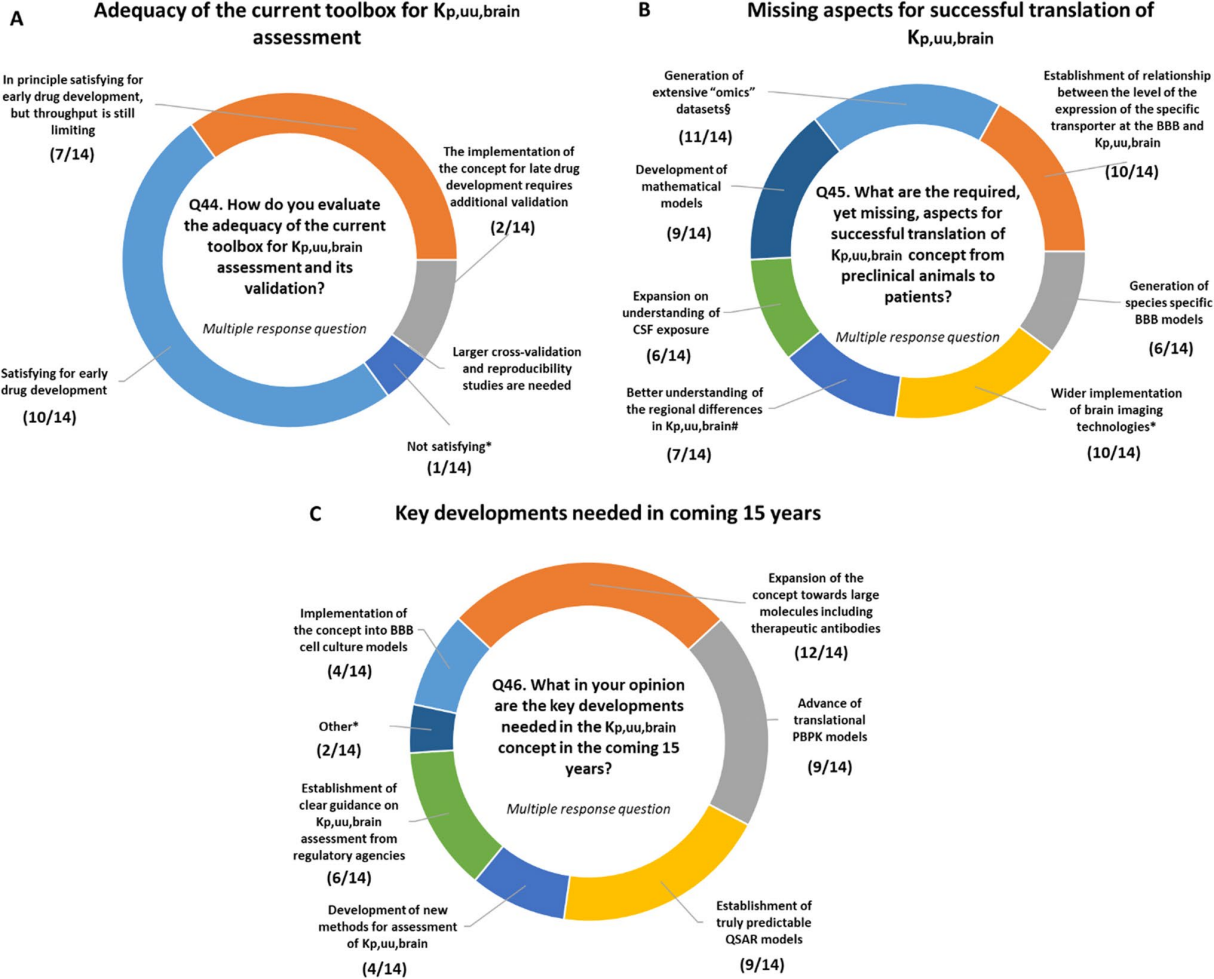
Evaluation of the adequacy of the current toolbox for assessment of K_p,uu,brain_ in pharmaceutical industry (**A**), required, yet missing, aspects for successful translation of K_p,uu,brain_ concept from preclinical animals to patients (**B**) and the key developments needed in the K_p,uu,brain_ concept in the coming 15 years (**C**). NB: In A Not satisfying*- We need a better understanding of unbound intracellular concentrations for the cells types of interest (both for efficacy and for safety); We need tools to explain/verify mechanistically unexpected K_p,uu_ values. In B §—Generation of extensive ‘omics’ datasets on interspecies differences in the expression of transporters at the BBB in healthy and pathological conditions; *—Wider implementation of translational brain imaging technologies (e.g. PET) in CNS drug development programs. In C Other*—Generation of additional K_p,uu_ data in higher species (e.g. monkeys, pigs) and human K_p,uu_ data. Better understanding of other efflux transporters besides P-gp and BCRP, and how these translate to humans; Impact of metabolism in the CNS; Regional differences; Novel uptake mechanisms.

Although, 10 out of 14 responders evaluate the adequacy of the current toolbox for K_p,uu,brain_ assessment and its validation as satisfactory for early drug development, seven responders indicate that the throughput is still a limiting factor (Fig. 6A). Only 2/14 specify that implementation of the concept for late drug development requires additional validation. Among the aspects that are considered to be non-satisfactory, responders mention a better understanding of unbound intracellular concentrations for the cells types of interest (both for efficacy and for safety) and a need for tools to explain/verify mechanistically unexpected K_p,uu,brain_ values.

It seems that pharmaceutical companies have rather similar opinions regarding the required, yet missing, aspects for successful translation of the K_p,uu,brain_ concept from preclinical species to patients (Fig. 6B). Generation of extensive ‘omics’ datasets on interspecies differences in the expression of transporters at the BBB in healthy and pathological conditions linked to establishment of a relationship between the level of the expression of the specific transporter at the BBB and K_p,uu,brain_ are thought to be one of the critical aspects. Understanding regional differences in K_p,uu,brain_ (also via deepening the knowledge employing ‘omics’ technologies) and their impact on the translation of data from preclinical systems also needs attention. Wider implementation of translational brain imaging technologies (e.g., PET) in CNS drug development programs has already been designated as an essential part for the translation.

When considering future perspectives related to the K_p,uu,brain_ concept, responders indicated the following areas that require attention in the coming 15 years (Fig. 6C):

- Expansion of the concept towards large molecules including therapeutic antibodies (12/14)
- Advance of translational PBPK models (9/14)
- Establishment of truly predictable QSAR models (9/14)
- Establishment of clear guidance on K_p,uu,brain_ assessment from regulatory agencies (6/14)

## Concluding Remarks

In the process of authoring this review, experts representing 15 pharmaceutical companies have come together to discuss and reflect upon the impact of the K_p,uu,brain_ concept in drug discovery and development. A story has emerged describing a grass-root driven implementation of the concept which has developed and matured into remarkably similar approaches between the companies, now strongly impacting on the workflows of drug design and translation to patient. Challenges ahead are generally recognized to connect with the lack of human K_p,uu,brain_ data to better understand the magnitude of impact of species differences in transporter expression and function. Hence, generation of additional K_p,uu,brain_ data in higher species (e.g. monkeys, pigs) and humans is critical. In this regard, an open exchange of all involved stakeholders (e.g., academia, industry, regulators) with regards to best practices, case examples and pitfalls would be invaluable. Education around the K_p,uu,brain_ concept has also been highlighted as one of the critical aspects. In fact, transfer of this knowledge to clinical development experts, clinicians as well as regulators may further facilitate CNS drug development still suffering from comparatively high attrition rates.

## Supplementary Information

Below is the link to the electronic supplementary material.

Supplementary file1 (DOCX 57 KB)

Supplementary file2 (PDF 242 KB)
