# Supplementary material for: Unbound Brain-to-Plasma Partition Coefficient, Kp,uu,brain—a Game Changing Parameter for CNS Drug Discovery and Development
Source: Pharm Res. 2022 Apr 11;39(7):1321–41. doi: 10.1007/s11095-022-03246-6 (PMC9246790; doi:10.1007/s11095-022-03246-6)
Supplement: Supplementary file 1 — Supplementary file1 (DOCX 57 KB) [file 11095_2022_3246_MOESM1_ESM.docx]

**S1. Questionnaire.**

Survey on the use/implementation of K_p,uu,brain_ concept by pharma companies

# Background questions

1. What is the size of your company?
2. ≤5,000 employees
3. >5,000 employees
4. Which department are you affiliated with?
   1. Drug Metabolism & Pharmacokinetics (DMPK)
   2. Pharmacology
   3. Clinical Pharmacology
   4. Medicinal Chemistry
   5. Other, please indicate.
5. What type of activities is your company mainly involved in?
   1. Drug discovery and development
   2. Contract research
   3. Consultancy
   4. Other, please specify
6. In which disease areas is your company mainly working? Select all that apply.
   1. Neuroscience
   2. Oncology
   3. Metabolic disorders
   4. Inflammation
   5. Cardiovascular
   6. Infectious disease
   7. Other, please specify
7. What drug modalities does your company work with? Select all that apply.
   1. Small molecules including PROTACs
   2. Peptides (<20 kDa)
   3. Protein therapeutics (>20 kDa)
   4. Nucleotide therapeutics
   5. Other
   6. I prefer not to disclose this information

# Implementation

1. When did the first project teams or key scientists start advocating for applying the concepts of the unbound brain-to-plasma concentration ratio (Kp,uu,brain)?
   1. Before 2000
   2. 2001-2005
   3. 2006-2010
   4. 2011-2015
   5. 2016-present
   6. Not applicable
2. When did your company begin to conduct the first experimental work towards estimating Kp,uu,brain?
   1. Before 2000
   2. 2001-2005
   3. 2006-2010
   4. 2011-2015
   5. 2016-present
   6. No experimental work has been conducted
3. What level of implementation and integration of Kp,uu,brain would you estimate in your company, as a % of project teams applying concepts and methodology as appropriate.
   1. Less than 20 %
   2. Between 20 and 80 %
   3. More than 80 %
4. When was the Kp,uu,brain concept fully embedded in the project teams? I.e. ca 80% of the projects applying concepts and methodology as appropriate.
   1. Before 2000
   2. 2001-2005
   3. 2006-2010
   4. 2011-2015
   5. 2016-present
   6. This level of embedding has not been yet reached
5. What was the main driver for introducing and implementing the Kp,uu,brain concept into your company? Select all that apply.
   1. Following general shift in paradigm and scientific rigour in pharmacology and pharmacokinetics
   2. Difficulties to explain what is the PK driver for efficacy
   3. Projects challenged with unexpected and unexplained CNS side effects
   4. Don´t know
6. What was the key mechanism by which Kp,uu,brain was implemented in you organisation?
   1. Portfolio decision from accountable function and co-ordinated roll out to project teams
   2. Provision of Kp,uu,brain data to a subset of projects as examples to prove usefulness of Kp,uu,brain concept
   3. Co-ordinated training of project teams in concepts of Kp,uu,brain
   4. Individual scientist advocating the application of Kp,uu,brain concepts in project teams
   5. Other, please specify.
7. Has your company implemented experimental estimation of Kp,uu,brain as a part of the default process (avoiding the need for specific project team decision) at any stage of screening or drug development?
   1. Yes, for every nominated drug candidate (e.g. prior to investment decision in GLP-tox studies)
   2. Yes, for every nominated drug candidate, but only for CNS projects (e.g. prior to investment decision in GLP-tox studies)
   3. Yes, Kp,uu,brain determination is part of our generic (company-wide) screening cascade and/or considered a prerequisite to conduct in vivo profiling (PK and/or PD)
   4. No, Kp,uu,brain is not included in any defined company-wide processes however most project teams will have developed their own process and criteria for conducting Kp,uu,brain determination.
   5. There is little such process
   6. Other, please specify.
8. Which department is primarily accountable for generating experimental data on Kp,uu,brain? Note: Other functions may be responsible for conducting elements of the work.
   1. Drug Metabolism & Pharmacokinetics (DMPK)
   2. Pharmacology
   3. Medicinal chemistry
   4. Clinical Pharmacology
   5. Other, please indicate.
9. Where is Kp,uu,brain (and its elements) experimentally determined?
   1. Mainly internally
   2. Mainly externally (by CRO or other collaborator)
   3. Internally and externally, depending on resources
   4. No response
   5. Other, please specify
10. In your view, is there generally, i. e. in 80% of projects, a common understanding/acceptance across disciplines in project teams (chemistry, DMPK and pharmacology) of the meaning and utility of Kp,uu,brain versus other metrics somehow relating to brain exposure?
11. Yes
12. No
13. With respect to project implementation of the Kp,uu,brain concept and methodology, what level of heterogeneity exists within your company between different therapeutic areas or geographical locations?
    1. There are significant differences due to e.g. tradition and/or preference of individual (groups of) scientists.
    2. There are differences. For the most part however, these can be seen to relate to the needs of the different therapy areas.
    3. The question does not apply to my company (e.g. too small).
    4. Other

# Application areas

1. Which of the following areas of application for Kp,uu,brain methodology are of impact and importance for project and portfolio progression? Select all that apply.
   1. Neuropharmacokinetic screening and profiling (from an efficacy standpoint). For instance using an estimate of Kp,uu,brain as gating criteria for entry into in vivo pharmacology studies.
   2. By successfully correlating measured Kp,uu,brain with in vitro assays (such assays efflux ratios), it has become possible to use this in vitro methodology (efficiently and reliably) to screen and identify and prioritize molecules with desired Kp,uu,brain.
   3. Definition of PK/PD relationship for CNS effects and/or prediction of therapeutic dose
   4. CNS off-target safety assessment
   5. CNS on-target safety assessment (e.g. the drug acts on a peripheral target which is also expressed in the brain, or a CNS target has additional functions which are sought to be avoided)
   6. Other, please add comment
2. Beyond rodents, in which other species is Kp,uu,brain determined (in vivo)? Select all that apply.
   1. Dogs
   2. NHP
   3. Pigs
   4. Humans
   5. Other, please specify
   6. None beyond rodents
3. What would be the drivers for inclusion of higher species (dog, NHP and human) in the assessment of Kp,uu,brain? Select all that apply.
   1. Reducing of uncertainty related to potential species differences for CNS targets
   2. Mitigating risk of CNS side-effects which are not easily monitorable in early clinical trials
   3. Better translation of human dose-exposure-CNS biomarker response relationships.
   4. Higher species (dog, NHP) are part of non-clinical safety/efficacy assessment.
   5. Does not apply as Kp,uu,brain is only determined in rodents
   6. Other, please specify and include reference to species
4. How does the numerical value of Kp,uu,brain (any species) feed into predictions of therapeutic dose (typically underpinning MABEL, starting dose, tox margins, or Phase 2 dose ranges etc)?
   1. Kp,uu,brain is not used in this context, or it only provides qualitative support for the approach taken in predicting therapeutic dose
   2. There are examples where we have used Kp,uu,brain as parameter with direct quantitative link to the estimate of therapeutic dose.
   3. Don´t know / no response
5. Do you use Kp,uu,brain for assessment of the effects of disease and age on transport across the BBB?
6. Yes, please add examples
7. No
8. Other, please add comment
9. Do you investigate drug-drug interactions on the level of BBB using Kp,uu,brain?
10. Yes
11. No
12. Other, please add comment
13. Are you addressing the intracellular exposure of brain parenchymal cells (neurons etc) by any means e.g. by measuring Kp,uu,brain,cell?
    1. No, we typically consider the brain interstitium (Kp,uu,brain) to adequately represent exposure in brain cells.
    2. Yes. Please specify rationale
    3. Other, please specify

# Methodology

1. Which in vivo methods are used at your company to determine Kp,uu,brain? Select all that apply.
   1. Brain/blood sampling over time with assessment of AUCs in preclinical animals followed by binding correction in respective tissue
   2. Brain/blood sampling at infusion steady-state in preclinical animals followed by binding correction in respective tissue
   3. Brain/blood sampling at a single non steady-state time-point in preclinical animals followed by binding correction in respective tissue
   4. Brain/blood microdialysis
   5. PET/SPECT brain imaging
   6. Other, please specify
2. Do you employ cassette dosing as means to increase throughput and reduce animal usage in the determination of Kp,uu,brain. Select which option that best describes your situation.
   1. Yes, in vivo cassette dosing is the primary means of obtaining Kp,uu,brain in rodents. Determination from animals dosed with single compound is typically limited to PD experiments, higher species studies or other exceptions.
   2. Sometimes, cassette dosing is used to support determination of Kp,uu,brain
   3. No, single compound dosing is typically used in rodents.
3. Which method is used for determination of brain tissue binding and uptake? Select all that apply.
   1. Equilibrium dialysis using brain homogenate
   2. Brain slice assay
   3. QSAR prediction
   4. Other method, please specify
4. Is brain free fraction using the brain homogenate method always measured in the same species as the in vivo study?
   1. Yes
   2. No, the company typically uses a single species and assumes species-independency of drug brain tissue binding properties. Please specify which species is used.
   3. Other, please specify
5. What elements of Kp,uu,brain methodology has been internally validated in your company? Select all that apply.
   1. Kp,uu,brain assessment using brain microdialysis
   2. Brain tissue uptake and binding assessment using brain homogenate vs brain slice assays
   3. Kp,uu,brain versus ratio of Kp,brain in e.g. mdr1a/b double knockout and wild type mice
   4. Validation against literature values of Kp,uu,brain
   5. Validation against literature values of fu,brain
   6. None of the above
   7. Other, please specify
6. Do you use BBB cell culture models to predict Kp,uu,brain? Select all that apply.
   1. Primary brain endothelial cells
   2. None brain endothelial cell lines (e.g. Caco-2, MDCK overexpressing efflux transporter such as MDR1 or BCRP)
   3. iPS cell lines
   4. No, we do not use any cell lines
   5. Other, please specify
7. Do you use in silico models to predict Kp,uu,brain? Select all that apply.
   1. Quantitative Structure Property Relationships (QSPR) derived by machine learning algorithms or other means. (Predictions from the chemical structure and/or chemical properties)
   2. Physiologically-based pharmacokinetic models for the brain tissue with parameters derived from chemical properties and/or in vitro assays.
   3. No, we do not use any in silico approach
   4. Other, please specify
8. Do you use CSF as a surrogate fluid to assess the free brain concentration?
   1. Yes, this is a common practice
   2. Sometimes (this is performed occasionally)
   3. No, this is essentially never done
   4. Other, please specify
9. Do project teams (continue to) ask to measure (total) brain exposure in addition to plasma concentrations in standard PK or PD studies?
   1. Yes, this is a common practice in at least some projects
   2. Yes, but only if concomitant biomarker responses are measured in the same tissue.
   3. No, brain tissue samples are typically only taken in the studies dedicated to estimate Kp,uu,brain.
10. For the purpose of determining Kp,uu,brain in pharmacology studies, do project teams typically conduct brain sampling of the animals in the study (including any satellite groups)?
    1. No, the estimate of Kp,uu,brain is obtained separately in a dedicated study and applied to measurement of plasma concentration to derive CNS exposure.
    2. Yes, it is generally routine to determine (total) brain concentrations across PD studies for future use.
    3. Other, please specify
11. In determining the temporal aspect of CNS exposure in pharmacology (e.g. unbound brain concentration at defined time-points such as pre-dose trough levels in repeated dosing studies) which of the of the following statements best reflects the way your company works:
    1. Brain exposure at specific time points is usually not considered for any major applications (including PK/PD and prediction of therapeutic dosage)
    2. It happens that there is a need or interest in assessing brain exposure at specific time points. In such instances we conduct sampling of brain at those timepoints to enable calculation of exposure.
    3. It happens that there is a need or interest in assessing brain exposure at specific time points. In such instances exposure is estimated from the plasma concentration and Kp,uu,brain determined based on AUC or at steady state (from the same study or separate dedicated study)
12. What other parameters (somehow related to Kp,uu) are being used for the equivalent purpose i.e. estimation CNS exposure or ‘extent of BBB transport’? Select all that apply.
    1. Endothelial permeability (e.g. in situ brain perfusion)
    2. CSF concentrations (e.g. Kp,uu,CSF)
    3. Total brain concentrations (including total brain-to-plasma ratio)
    4. fu,brain (as a standalone parameter)
    5. fu,plasma (as a standalone parameter)
    6. Other, please specify
13. Do you have established values for Kp,uu,brain which are considered as “good” or “bad” ?
    1. Yes, we have such values established and use them for compound prioritization. Please specify specific Kp,uu,brain value the projects/organization would consider for a compound being ‘progressable’
    2. No
    3. Other, please specify
14. Do you use transgenic animal lines for clarification of the certain BBB mechanism and verify the Kp,uu,brain?
15. Yes
16. No
17. Other, please specify
18. Do you apply acceptance criteria for Kp,uu,brain, positive or negative controls to assess data quality/consistency?
    1. No
    2. Yes
    3. If Yes, provide a brief summary
19. How do you interpret and report development data for submission to regulatory authorities that are based on total brain concentrations, such as Quantitative whole body autoradiography (QWBA) data?

Please describe as applicable.

1. Have you ever reported Kp,uu,brain data to regulatory agency?
2. No, not that I am aware of.
3. Yes ,please specify

# Impact

1. How would you rate and describe the portfolio impact of Kp,uu,brain implementation in your company? Please select one statement that best corresponds to your view.
   1. Implementation of Kp,uu,brain has been game-changing and I know of several examples where Kp,uu,brain methodology has enabled or accelerated project progression by changing the course of chemical series development, or enabled critical understanding of CNS PK/PD.
   2. Implementation of Kp,uu,brain concept and methods is scientifically justified and have facilitated the ways we work and communicate in a positive way. There is likely also positive portfolio impact here, but (despite my experience) I would struggle to pull out examples where it has factually made a difference for a project versus using other metrics or not having them at all.
   3. Implementation of Kp,uu,brain is perhaps theoretically correct (who really knows about these unbound concentrations), but it has mainly meant more work for us with unclear benefit.
   4. Other, please specify
2. In which areas has Kp,uu,brain implementation impacted in such a clear way that you would be able to give a qualified example if asked to do so? Check all that apply.
   1. Better understanding of PK/PD relationships (versus if one had not had the methods) have helped us to define tactics of project progression or benchmarking.
   2. We have evidence/examples of how Kp,uu,brain methodology has enabled more appropriate selection of compounds for progression.
   3. The Kp,uu,brain value is used in such a way that it directly impacts estimation of therapeutic dose and activities that are tied to that dose estimate.
   4. Other, please describe.
3. How has the implementation of the of Kp,uu,brain concept and methodology impacted the number of experimental animals used? Select the statements which best represents your view.
4. Measurement of Kp,uu,brain requires additional animals and has therefore increased the use of experimental animals.
5. By enabling better selection of molecules to enter in vivo testing we have been able to reduce the use of animals.
6. There is no clear impact on the number of animals used for each project in isolation. However by enabling better selection of compounds it has increased the probability of success and can therefore be seen as a reduction in animal use.
7. None of the above

# Future perspectives

1. How do you evaluate the adequacy of the current toolbox for Kp,uu,brain assessment and its validation? Select all that apply.
   1. Satisfying for early drug development
   2. In principle satisfying for early drug development, but throughput is still limiting
   3. The implementation of the concept for late drug development requires additional validation
   4. Larger cross-validation and reproducibility studies are needed
   5. Not satisfying, please specify
2. What are the required, yet missing, aspects for successful translation of Kp,uu,brain concept from preclinical animals to patients? Select all that apply.
   1. Generation of extensive “omics” datasets on interspecies differences in the expression of transporters at the BBB in healthy and pathological conditions
   2. Establishment of relationship between the level of the expression of the specific transporter at the BBB and Kp,uu,brain
   3. Generation of species specific BBB models and estimation of Kp,uu,brain
   4. Wider implementation of translational brain imaging technologies (e.g. PET) in CNS drug development programs
   5. Better understanding of the regional differences in Kp,uu,brain and how that impacts the translation of data from preclinical systems
   6. Expansion on understanding of CSF exposure
   7. Development of mathematical models (e.g. PBPK(PD), scaling of PK/PD models)
   8. Other, please specify
3. What in your opinion are the key developments needed in the Kp,uu,brain concept in the coming 15 years. Select all that apply.
   1. Implementation of the concept into BBB cell culture models
   2. Expansion of the concept towards large molecules including therapeutic antibodies
   3. Advance of translational PBPK models
   4. Establishment of truly predictable QSAR models
   5. Development of new methods for assessment of Kp,uu,brain
   6. Establishment of clear guidance on Kp,uu,brain assessment from regulatory agencies
   7. Other, please specify
4. In my opinion the question related to Kp,uu,brain that needs the most attention is:

Please add free text.
