# Supplementary material for: Unbound Brain-to-Plasma Partition Coefficient, Kp,uu,brain—a Game Changing Parameter for CNS Drug Discovery and Development
Source: Pharm Res. 2022 Apr 11;39(7):1321–41. doi: 10.1007/s11095-022-03246-6 (PMC9246790; doi:10.1007/s11095-022-03246-6)
Supplement: Supplementary file 2 — Supplementary file2 (PDF 242 KB) [file 11095_2022_3246_MOESM2_ESM.pdf]

Sammanställning av Survey on the use/implementation of Kp,uu,brain concept by pharma companies

The survey is consisting of 47 questions related to Kp,uu,brain, falling into the following categories:

1. Background questions 2. Implementation 3. Application areas 4. Methodology 5. Impact 6. Future perspective

The survey will be open on 22nd of September and end on 3rd of October.

The summary of the survey generated automatically by the KURT system will be circulated in PDF and Excel formats by 4th of October. After discussion, the results will be used in the preparation of the perspective paper for an honorary special issue of Pharmaceutical Research (PharmRes®), dedicated to Professor Emerita Margareta Hammarlund-Udenaes.

NB: The survey is anonymous.

Sammanställd	
Antal svar	14
Tillgänglig	2021-09-21 – 2021-10-14
Kontaktperson	Irena Loryan (irena.loryan@farmaci.uu.se), verksam vid tPKPD group

1. What is the size of your company?

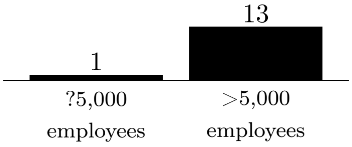

2. Which department are you affiliated with?

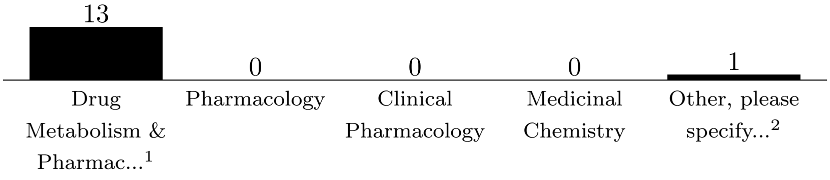

[1] Drug Metabolism & Pharmacokinetics (DMPK)
[2] Other, please specify

Other, please specify: External Innovation (R&D)

3. What type of activities is your company mainly involved in?

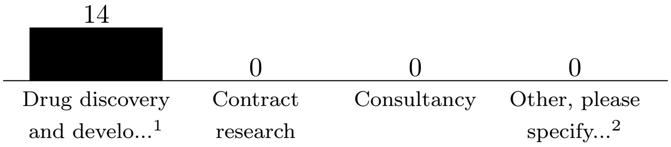

[1] Drug discovery and development
[2] Other, please specify

4. In which disease areas is your company mainly working? Select all that apply.

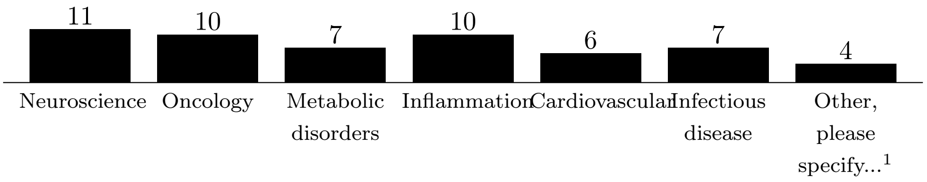

[1] Other, please specify

OTHER, PLEASE SPECIFY: Rare diseases, Pain and rare disease, Rare blood disorders; rare endocrine disorders, Pulmonary

5. What drug modalities does your company work with? Select all that apply.

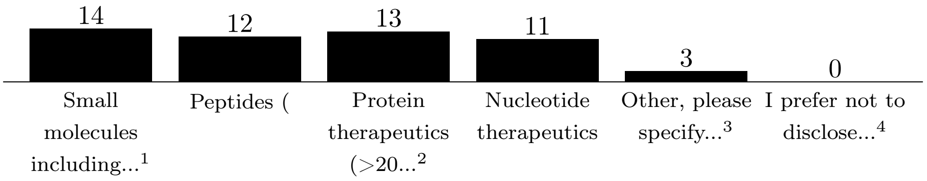

[1] Small molecules including PROTACs
[2] Protein therapeutics (>20 kDa)
[3] Other, please specify
[4] I prefer not to disclose this information

OTHER, PLEASE SPECIFY: Vaccine, gene therapy, Gene therapies (if not considered a part of "nucleotide therapeutics")

6. When did the first project teams or key scientists start advocating for applying the concepts of the unbound brain-to-plasma concentration ratio (Kp,uu,brain)?

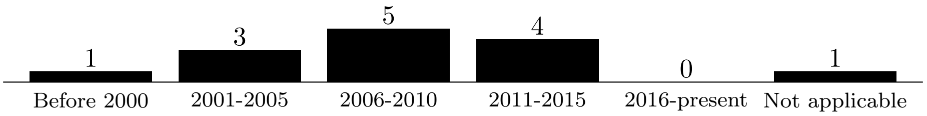

7. When did your company begin to conduct the first experimental work towards estimating Kp,uu,brain?

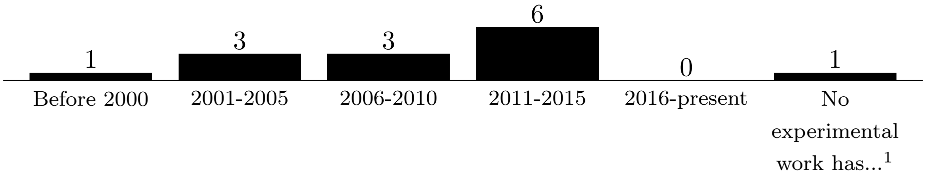

[1] No experimental work has been conducted

8. What level of implementation and integration of Kp,uu,brain would you estimate in your company, as a % of project teams applying concepts and methodology as appropriate.

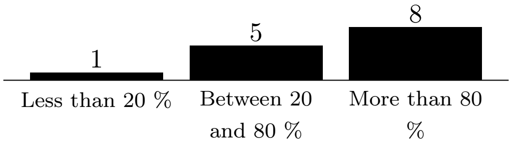

9. When was the Kp,uu,brain concept fully embedded in the project teams? I.e. ca 80% of the projects applying concepts and methodology as appropriate.

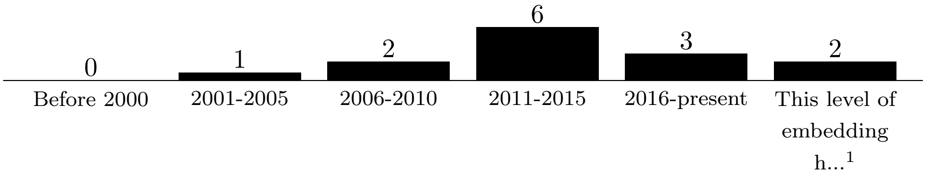

1 This level of embedding has not been yet reached

10. What was/were the main driver(s) for introducing and implementing the Kp,uu,brain concept into your company? Select all that apply.

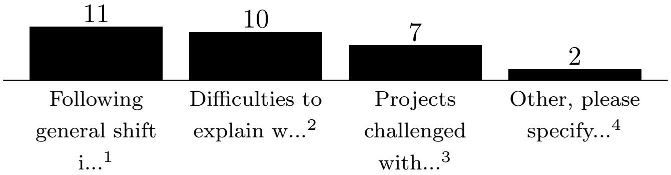

1 Following general shift in paradigm and scientific rigour in pharmacology and pharmacokinetics
2 Difficulties to explain what is the PK driver for efficacy
3 Projects challenged with unexpected and unexplained CNS side effects
4 Other, please specify

OTHER, PLEASE SPECIFY: Not yet implemented, Application of free drug hypothesis also for targets within the CNS, both on- and off-targets

11. What was the key mechanism by which Kp,uu,brain was implemented in your organisation?

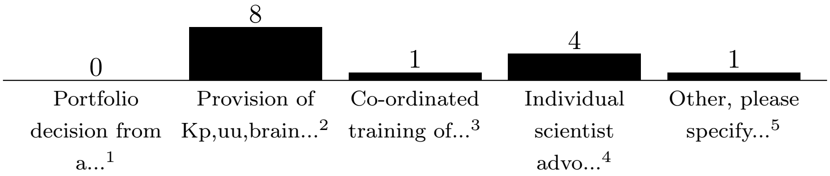

1 Portfolio decision from accountable function and co-ordinated roll out to project teams
2 Provision of Kp,uu,brain data to a subset of projects as examples to prove usefulness of Kp,uu,brain concept
3 Co-ordinated training of project teams in concepts of Kp,uu,brain
4 Individual scientist advocating the application of Kp,uu,brain concepts in project teams
5 Other, please specify

OTHER, PLEASE SPECIFY: Not yet implemented

12. Has your company implemented experimental estimation of Kp,uu,brain as a part of the default process (avoiding the need for specific project team decision) at any stage of screening or drug development?

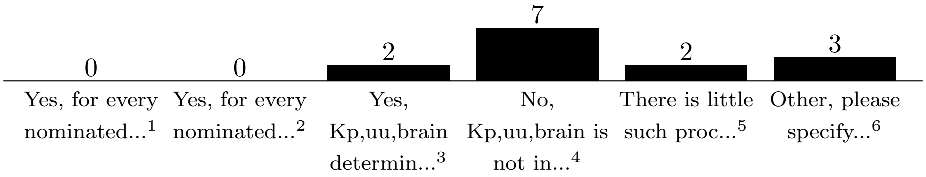

[1] Yes, for every nominated drug candidate (e.g. prior to investment decision in GLP-tox studies)
[2] Yes, for every nominated drug candidate, but only for CNS projects (e.g. prior to investment decision in GLP-tox studies)
[3] Yes, Kp,uu,brain determination is part of our generic (company-wide) screening cascade and/or considered a prerequisite to conduct in vivo profiling (PK and/or PD)
[4] No, Kp,uu,brain is not included in any defined company-wide processes however most project teams will have developed their own process and criteria for conducting Kp,uu,brain determination
[5] There is little such process
[6] Other, please specify

OTHER, PLEASE SPECIFY: Answer c), but only for CNS projects . ?Only for CNS projects? applies also to question 8., Informal process for projects that are requiring brain exposure utilizing CNS strategy, Yes, for projects where CNS penetration and unbound brain exposure is a critical parameter e.g. for on-target efficacy, for on-target central side-effects, for off-target central side-effects

13. Which department is primarily accountable for generating experimental data on Kp,uu,brain? Note: Other functions may be responsible for conducting elements of the work.

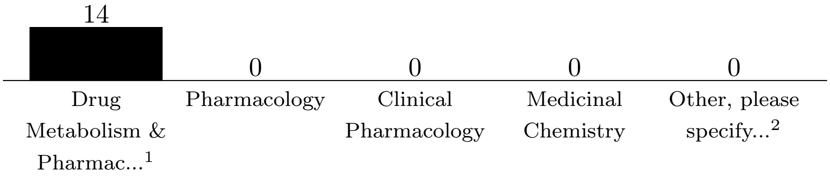

[1] Drug Metabolism & Pharmacokinetics (DMPK)
[2] Other, please specify

14. Where is Kp,uu,brain (and its elements) experimentally determined?

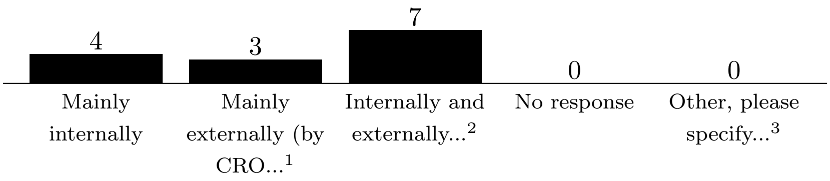

[1] Mainly externally (by CRO or other collaborator)
[2] Internally and externally, depending on resources
[3] Other, please specify

15. In your view, is there generally, i. e. in 80% of projects, a common understanding/acceptance across disciplines in project teams (chemistry, DMPK and pharmacology) of the meaning and utility of Kp,uu,brain versus other metrics somehow relating to brain exposure?

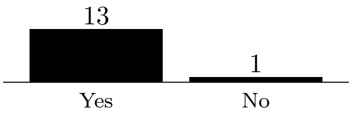

16. With respect to project implementation of the Kp,uu,brain concept and methodology, what level of heterogeneity exists within your company between different therapeutic areas or geographical locations?

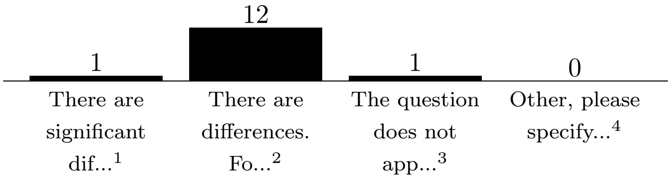

[1] There are significant differences due to e.g. tradition and/or preference of individual (groups of) scientists.
[2] There are differences. For the most part however, these can be seen to relate to the needs of the different therapy areas.
[3] The question does not apply to my company (e.g. too small).
[4] Other, please specify

17. Which of the following areas of application for Kp,uu,brain methodology are of impact and importance for project and portfolio progression. Select all that apply.

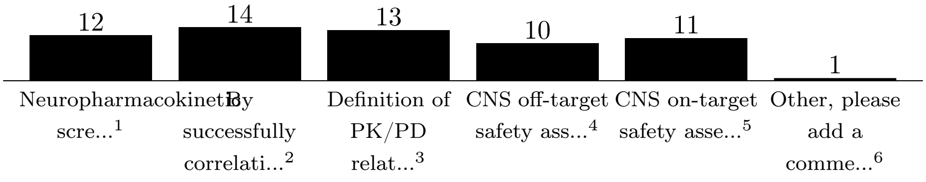

[1] Neuropharmacokinetic screening and profiling (from an efficacy standpoint). For instance using an estimate of Kp,uu,brain as gating criteria for entry into in vivo pharmacology studies.
[2] By successfully correlating measured Kp,uu,brain with in vitro assays (such assays efflux ratios), it has become possible to use this in vitro methodology (efficiently and reliably) to screen and identify and prioritize molecules with desired Kp,uu,brain.
[3] Definition of PK/PD relationship for CNS effects and/or prediction of therapeutic dose
[4] CNS off-target safety assessment
[5] CNS on-target safety assessment (e.g. the drug acts on a peripheral target which is also expressed in the brain, or a CNS target has additional functions which are sought to be avoided)
[6] Other, please add a comment

OTHER, PLEASE ADD A COMMENT: In addition to the absolute Kp,uu value, project teams apply the unbound brain concentrations derived from a Kp,uu experiment to assess theoretical target coverage (e.g. Cu,br (nM) vs in vitro target IC50 (nM)). Hence a compound with a sub-optimal Kp,uu (e.g. 0.2) may prove progressable if sufficient unbound brain concentrations can be achieved to engage target (permitting peripheral side effect/TI profile)

18. Beyond rodents, in which other species is Kp,uu,brain determined (in vivo)? Select all that apply.

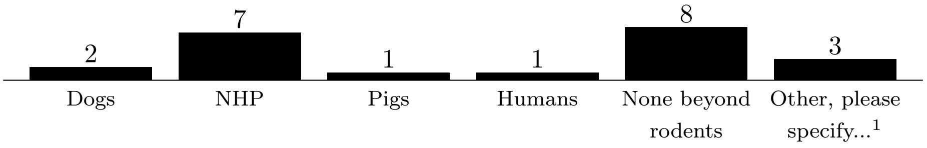

[1] Other, please specify

Other, please specify: non-rodents only at late discovery stage (close to candidate selection) to strengthen human translation, There may occasionally be PET data used to calculate Kpuu in NHP and humans, NHP PET receptor occupancy data along with other data is used to infer NHP Kp,uu,brain, but Kp,uu,brain has not been measured directly from NHP.

19. What would be the drivers for inclusion of higher species (dog, NHP and human) in the assessment of Kp,uu,brain? Select all that apply.

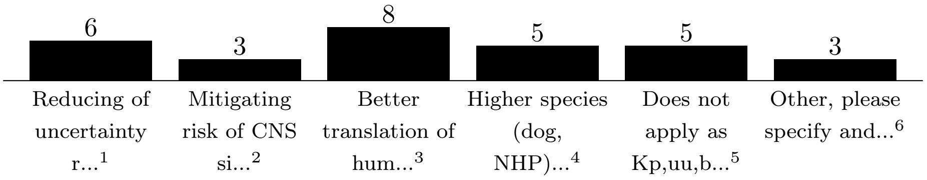

[1] Reducing of uncertainty related to potential species differences for CNS targets
[2] Mitigating risk of CNS side-effects which are not easily monitorable in early clinical trials
[3] Better translation of human dose-exposure-CNS biomarker response relationships
[4] Higher species (dog, NHP) are part of non-clinical safety/efficacy assessment
[5] Does not apply as Kp,uu,brain is only determined in rodents
[6] Other, please specify and include reference to species

Other, please specify and include reference to species: Mitigation of species dependent transporter mediated efflux (eg. BCRP in monkey), Kp,uu,brain in other species is being rationally approached rather than experimentally determined, typically applying all relevant contextual data, NHP PET receptor occupancy data along with other data is used to infer NHP Kp,uu,brain, but Kp,uu,brain has not been measured directly from NHP.

20. How does the numerical value of Kp,uu,brain (any species) feed into predictions of therapeutic dose (typically underpinning MABEL, starting dose, tox margins, or Phase 2 dose ranges etc)?

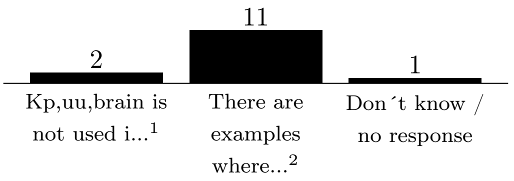

[1] Kp,uu,brain is not used in this context, or it only provides qualitative support for the approach taken in predicting therapeutic dose.
[2] There are examples where we have used Kp,uu,brain as parameter with direct quantitative link to the estimate of therapeutic dose.

21. Do you use Kp,uu,brain for assessment of the effects of disease and age on transport across the BBB?

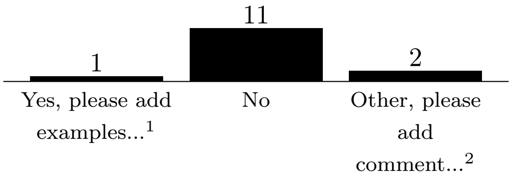

[1] Yes, please add examples
[2] Other, please add comment

Other, please add comment: Kp,uu have been determined in specific pharmacological animal models that may influence BBB permeation (e.g. drug induced seizure animal model or trangsgenic mice models for neurological targets e.g. alzheimers)

Yes, please add examples: Yes, we did it once for a target from which we know that the expression and the final pharmacological effect is age dependent in mice. The compound was a moderate P-gp substrate but there was no difference in Kp,uu,brain on age in mice. We never tested it for a strong P-gp substrate.

22. Do you investigate drug-drug interactions on the level of BBB using Kp,uu,brain?

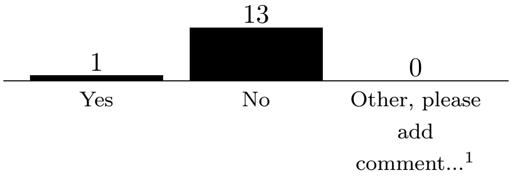

[1] Other, please add comment

23. Are you addressing the intracellular exposure of brain parenchymal cells (neurons etc) by any means e.g. by measuring Kp,uu,brain,cell?

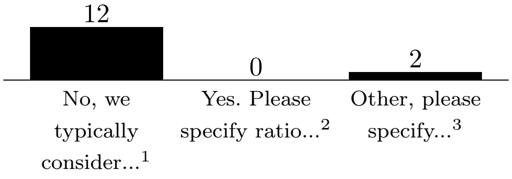

[1] No, we typically consider the brain interstitium (Kp,uu,brain) to adequately represent exposure in brain cells
[2] Yes. Please specify rationale
[3] Other, please specify

Other, please specify: Answer a) for Research, answer b) for Development, here we use calculated Kp,uu,cell to correct Fubrain from brain homogenate for lysosomal uptake and pH differences to derive a better estimation of Kp,uu,brain., Kp,uu,cell is assessed for in vitro cell models (tumor cell lines, hepatocyetes) and other tissues (liver) but not for brain.

24. Which in vivo methods are used at your company to determine Kp,uu,brain? Select all that apply.

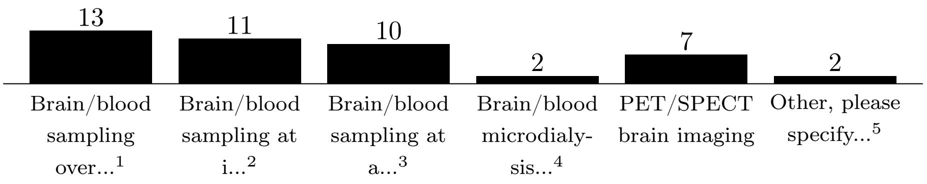

[1] Brain/blood sampling over time with assessment of AUCs in preclinical animals followed by binding correction in respective tissue
[2] Brain/blood sampling at infusion steady-state in preclinical animals followed by binding correction in respective tissue
[3] Brain/blood sampling at a single non steady-state time-point in preclinical animals followed by binding correction in respective tissue
[4] Brain/blood microdialysis
[5] Other, please specify

Other, please specify: Plans to do PET imaging for compounds in Development, e.g. for compounds targeting brain tumors., Not yet broadly implemented

25. Do you employ cassette dosing as means to increase throughput and reduce animal usage in the determination of Kp,uu,brain. Select which option that best describes your situation.

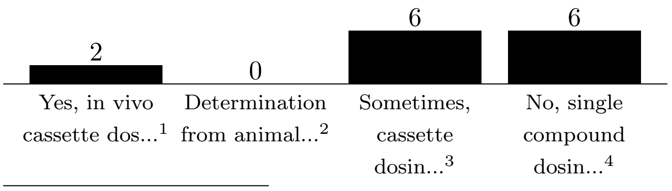

[1] Yes, in vivo cassette dosing is the primary means of obtaining Kp,uu,brain in rodents
[2] Determination from animals dosed with single compound is typically limited to PD experiments, higher species studies or other exceptions
[3] Sometimes, cassette dosing is used to support determination of Kp,uu,brain
[4] No, single compound dosing is typically used in rodents

26. Which method is used for determination of brain tissue binding and uptake? Select all that apply.

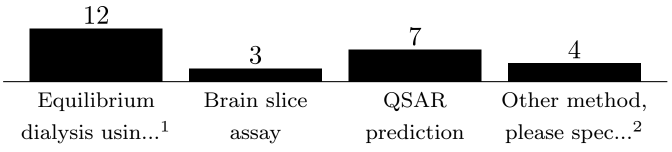

[1] Equilibrium dialysis using brain homogenate
[2] Other method, please specify

Other method, please specify: Muscle to brain ratio to estimate efflux at the BBB (Pharmaceutics, 11, 595 (2019), doi 10.3390/pharmaceutics11110595)., 96-well filter membrane impregnated with brain homogenate. The assay is called LIMBA (Lipid membrane binding assay). This assay is alos used to optimize against a high non-specific binding in the development of PET tracers. doi: 10.1016/j.ejps.2015.08.014, Imaging, Ultracentrifugation, ultrafiltration

27. Is brain free fraction using the brain homogenate method always measured in the same species as the in vivo study?

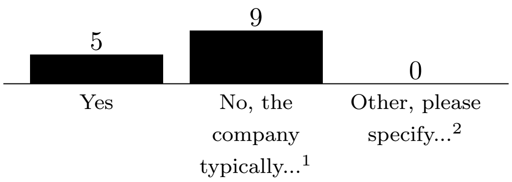

[1] No, the company typically uses a single species and assumes species-independency of drug brain tissue binding properties. Please specify which species is used.
[2] Other, please specify

NO, THE COMPANY TYPICALLY USES A SINGLE SPECIES AND ASSUMES SPECIES-INDEPENDENCY OF DRUG BRAIN TISSUE BINDING PROPERTIES. PLEASE SPECIFY WHICH SPECIES IS USED.: Rat, We use porcine brain polar lipids. Correlates very well with brain homogenates from rat, mouse, monkey, human brain., rat, rat, rat brain homogenate, Rat

28. What elements of Kp,uu,brain methodology has been internally validated in your company? Select all that apply.

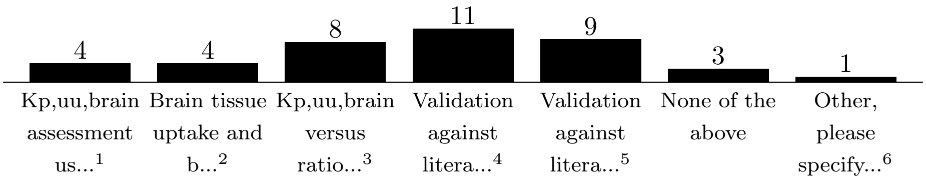

[1] Kp,uu,brain assessment using brain microdialysis
[2] Brain tissue uptake and binding assessment using brain homogenate vs brain slice assays
[3] Kp,uu,brain versus ratio of Kp,brain in e.g. mdr1a/b double knockout and wild type mice
[4] Validation against literature values of Kp,uu,brain
[5] Validation against literature values of fu,brain
[6] Other, please specify

OTHER, PLEASE SPECIFY: validation in characterizing cross compound and cross series relationship between unbound brain concentration and pharmacodynamic responses measured preclinically.

29. Do you use BBB cell culture models to predict Kp,uu,brain? Select all that apply.

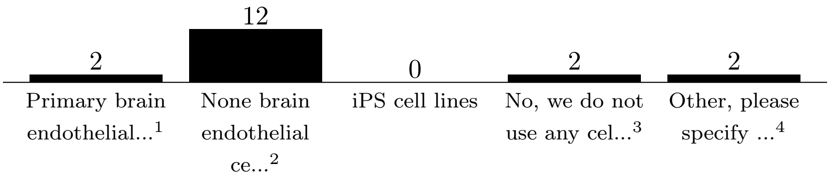

[1] Primary brain endothelial cells
[2] None brain endothelial cell lines (e.g. Caco-2, MDCK overexpressing efflux transporter such as MDR1 or BCRP)
[3] No, we do not use any cell lines
[4] Other, please specify

OTHER, PLEASE SPECIFY : No absolute prediction of Kp,uu performed from cell studies; rather qualitative information around in vitro - in vivo efflux correlations, we use LLC-PK1 cell line

30. Do you use in silico models to predict Kp,uu,brain? Select all that apply.

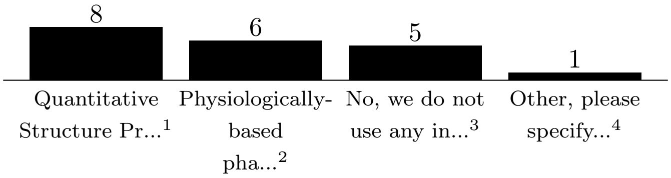

[1] Quantitative Structure Property Relationships (QSPR) derived by machine learning algorithms or other means. (Predictions from the chemical structure and/or chemical properties)
[2] Physiologically-based pharmacokinetic models for the brain tissue with parameters derived from chemical properties and/or in vitro assays
[3] No, we do not use any in silico approach
[4] Other, please specify

OTHER, PLEASE SPECIFY: We've developed individual in-house in silico models for fu,brain, fu,plasma, MDCK P-gp efflux ratio, and passive permeability although these separate models have not been combined together and validated for ability to predicted Kp,uu,brain

31. Do you use CSF as a surrogate fluid to assess the free brain concentration?

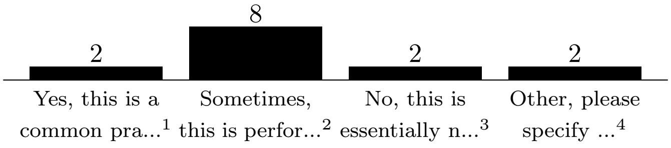

[1] Yes, this is a common practice
[2] Sometimes, this is performed occasionally
[3] No, this is essentially never done
[4] Other, please specify

OTHER, PLEASE SPECIFY : CSF only used for large molecule (mAbs) brain exposure assessment, Answer b), but only for compounds which are no transporter substrates.

32. Do project teams (continue to) ask to measure (total) brain exposure in addition to plasma concentrations in standard PK or PD studies?

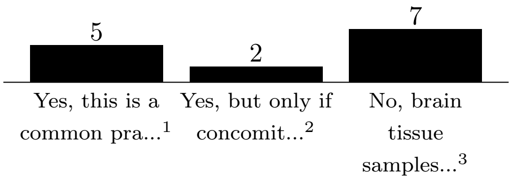

[1] Yes, this is a common practice in at least some projects
[2] Yes, but only if concomitant biomarker responses are measured in the same tissue
[3] No, brain tissue samples are typically only taken in the studies dedicated to estimate Kp,uu,brain

33. For the purpose of determining Kp,uu,brain in pharmacology studies, do project teams typically conduct brain sampling of the animals in the study (including any satellite groups).

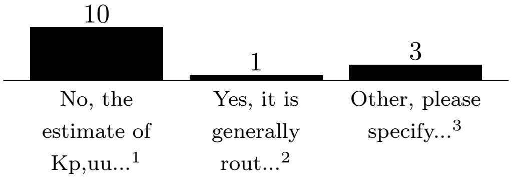

[1] No, the estimate of Kp,uu,brain is obtained separately in a dedicated study and applied to measurement of plasma concentration to derive CNS exposure

[2] Yes, it is generally routine to determine (total) brain concentrations across PD studies for future use

[3] Other, please specify

OTHER, PLEASE SPECIFY: Sometimes; depends on PD model/# animals, stagegate of compound(s), etc., Not yet broadly implemented, Sometimes estimate of Kp,uu,brain comes from separate dedicated "PK" study and applied to measurements of plasma concentration to derive CNS exposure, other times brain concentration is determined from PD studies for KPuu,brain determination and PK/PD analysis

34. In determining the temporal aspect of CNS exposure in pharmacology (e.g. unbound brain concentration at defined time-points such as pre-dose trough levels in repeated dosing studies) which of the of the following statements best reflects the way your company works:

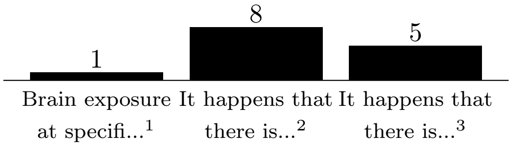

[1] Brain exposure at specific time points is usually not considered for any major applications (including PK/PD and prediction of therapeutic dosage).

[2] It happens that there is a need or interest in assessing brain exposure at specific time points. In such instances we conduct sampling of brain at those timepoints to enable calculation of exposure.

[3] It happens that there is a need or interest in assessing brain exposure at specific time points. In such instances exposure is estimated from the plasma concentration and Kp,uu,brain determined based on AUC or at steady state (from the same study or separate dedicated study).

35. What other parameters (somehow related to Kp,uu) are being used for the equivalent purpose i.e. estimation CNS exposure or ?extent of BBB transport?? Select all that apply.

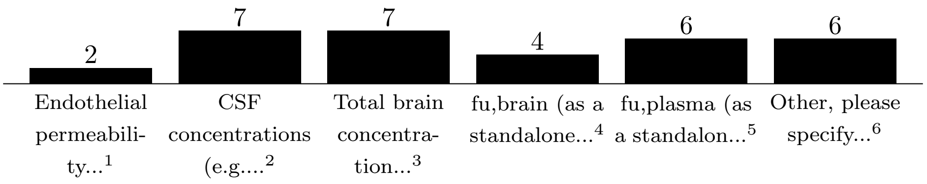

[1] Endothelial permeability (e.g. in situ brain perfusion)
[2] CSF concentrations (e.g. Kp,uu,CSF)
[3] Total brain concentrations (including total brain-to-plasma ratio)
[4] fu,brain (as a standalone parameter)
[5] fu,plasma (as a standalone parameter)
[6] Other, please specify

Other, please specify: Efflux ratio in transfected cells of MDR1 and BCRP, Total brain exposure is always used together with fu,brain, Muscle to brain ratio (Pharmaceutics, 11, 595 (2019), doi 10.3390/pharmaceutics11110595), in vivo receptor occupancy studies, Imaging, None of the above

36. Do you have established values for Kp,uu,brain which are considered as ?good? or ?bad? ?

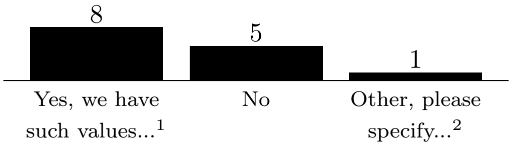

[1] Yes, we have such values established and use them for compound prioritization. Please specify specific Kp,uu,brain value the projects/organization would consider for a compound being ?progressable?.
[2] Other, please specify

Yes, we have such values established and use them for compound prioritization. Please specify specific Kp,uu,brain value the projects/organization would consider for a compound being ?progressable?.: generally 0.3, but can be project dependent., Our aspirational cut-off for a candidate molecule to move into the clinic is >0.5. For early leads deemed progressable, a cut-off of 0.3 is typically applied. There may be specific projects where the pursued target give rise adjusted cut-offs (e.g. high risk of peripheral side effects)., case by case, depends on the team (either >0.3 or >0.5), typically 0.5-2, but where PD response is observed, it may go outside this range (e.g. lower), >0.3 is good CNS exposure, >0.3, Cutoff values are specific to a given project, whether ?good? brain penetration is warranted (or not warranted) and expected impact on therapeutic index and/or dosing regimen. Any value of Kp,uu,brain that allows an acceptable therapeutic index and dosing regimen is considered an acceptable Kp,uu,brain. Generally, cut-off value of >0.3 defines ?good? brain penetration and value

Other, please specify: Values are defined for ”likely equilibrium”, but all other values ”depend” on the specific situation/molecule.

37. Do you use transgenic animal lines for clarification of the certain BBB mechanism and verify the Kp,uu,brain?

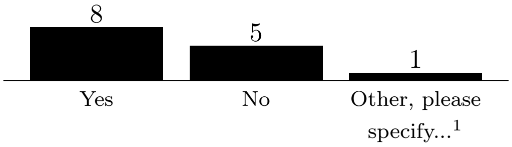

[1] Other, please specify

Other, please specify: Knock-out animals to verify involvement of efflux transporter

38. Do you apply acceptance criteria for Kp,uu,brain, positive or negative controls to assess data quality/consistency?

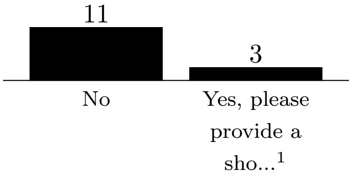

[1] Yes, please provide a short summary

Yes, please provide a short summary: Kp,uu determined in 3 rats - standard deviation is assessed and should fall within limits for data to be aggregated and reported as a mean in the database, Aceptance criteria are based on quality of the in vivo and in vitro experimental data and from the context of all that is known in the project

39. How do you interpret and report development data for submission to regulatory authorities that are based on total brain concentrations, such as Quantitative whole body autoradiography (QWBA) data? Please describe as applicable.

- QWBA is qualitative and signals maybe be contaminated by metabolites. If not intensity in the brain, suggesting poor brain penetration.
- Reported as total concentrations for regulatory purposes
- We do not use QWBA data for Kpuu assessesment because data represent total drug related radioactivity (parent plus potential metabolites), i.e. not only parent drug.
- We don't use QWBA results to draw any conclusion about the brain penetration of a compound
- As qualitative yes or no CNS penetration, since metabolite and parent are not separated in QWBA study
- Yes - we report a Kp and fu,b typically, sometimes Kp,uu. QWBA data are reported as such - no specific call-out to brain.
- Primarily imaging data has been used and reported semi-quantitatively
- not applicable
- typically only when negative, where there is a lack of compound in the brain
- These data are used for prediction of committed effective dose in human
- Images shared
- Report QWBA
- Such data are only interpreted at the level of total exposures
- Quantitative whole body autoradiography (QWBA) data and in vitro efflux data

40. Have you ever reported Kp,uu,brain data to regulatory agency?

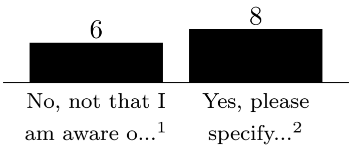

[1] No, not that I am aware of
[2] Yes, please specify

YES, PLEASE SPECIFY: PK/PD modelling, pharmacological effects and tox., During the filing process of drug candidates to show the regulatory agency that, the compound is not subject to active transport in vivo at the level of the BBB., Kpuu studies written up as non clinical study reports in tissue distribution and data included in IB and IND documents, Reported to demonstrate equilibrium at the BBB across species and its relevance to humans for a tight TI compound., it has been reported in Investigator Brochures and submissions, When relevant, This information goes into drug distribution section of IB, so technically gets reported to all agencies, as part of the prediction of the unbound brain exposure

41. How would you rate and describe the portfolio impact of Kp,uu,brain implementation in your company? Please select one statement that best corresponds to your view.

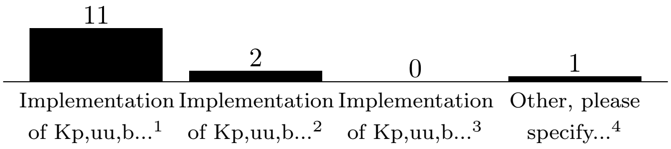

[1] Implementation of Kp,uu,brain has been game-changing and I know of several examples where Kp,uu,brain methodology has enabled or accelerated project progression by changing the course of chemical series development, or enabled critical understanding of CNS PK/PD
[2] Implementation of Kp,uu,brain concept and methods is scientifically justified and have facilitated the ways we work and communicate in a positive way. There is likely also positive portfolio impact here, but (despite my experience) I would struggle to pull out examples where it has factually made a difference for a project versus using other metrics or not having them at all
[3] Implementation of Kp,uu,brain is perhaps theoretically correct (who really knows about these unbound concentrations), but it has mainly meant more work for us with unclear benefit
[4] Other, please specify

OTHER, PLEASE SPECIFY: We haves struggled with its implementation and are still at stage where its implementation is still in its infancy

42. In which areas has Kp,uu,brain implementation impacted in such a clear way that you would be able to give a qualified example if asked to do so? Check all that apply.

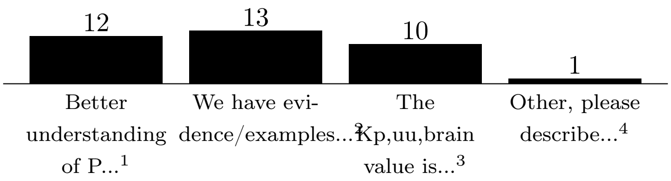

[1] Better understanding of PK/PD relationships (versus if one had not had the methods) have helped us to define tactics of project progression or benchmarking
[2] We have evidence/examples of how Kp,uu,brain methodology has enabled more appropriate selection of compounds for progression
[3] The Kp,uu,brain value is used in such a way that it directly impacts estimation of therapeutic dose and activities that are tied to that dose estimate
[4] Other, please describe

OTHER, PLEASE DESCRIBE: Not yet sufficient examples

43. How has the implementation of the of Kp,uu,brain concept and methodology impacted the number of experimental animals used? Select the statements which best represents your view.

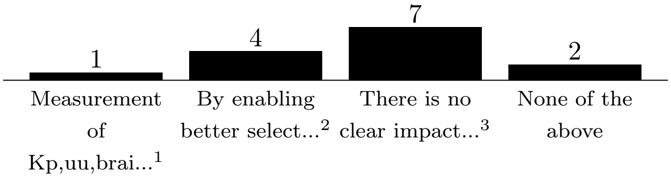

[1] Measurement of Kp,uu,brain requires additional animals and has therefore increased the use of experimental animals.
[2] By enabling better selection of molecules to enter in vivo testing we have been able to reduce the use of animals.
[3] There is no clear impact on the number of animals used for each project in isolation. However by enabling better selection of compounds it has increased the probability of success and can therefore be seen as a reduction in animal use.

44. How do you evaluate the adequacy of the current toolbox for Kp,uu,brain assessment and its validation? Select all that apply.

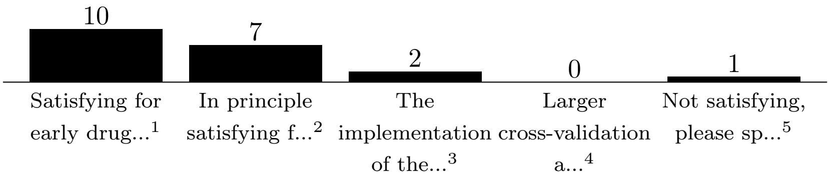

[1] Satisfying for early drug development
[2] In principle satisfying for early drug development, but throughput is still limiting
[3] The implementation of the concept for late drug development requires additional validation
[4] Larger cross-validation and reproducibility studies are needed
[5] Not satisfying, please specify

NOT SATISFYING, PLEASE SPECIFY: - We need a better understand of unbound intracellular concentrations for the cells types of interest (both for efficacy and for safety); - We need tools to explain/verify mechanistically unexpected Kp,uu values

45. What are the required, yet missing, aspects for successful translation of Kp,uu,brain concept from preclinical animals to patients? Select all that apply.

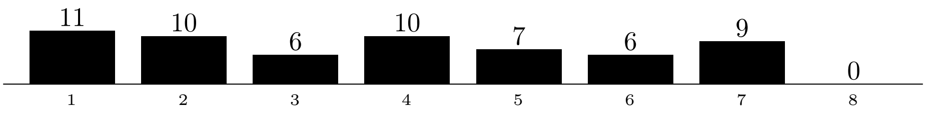

[1] Generation of extensive ?omics? datasets on interspecies differences in the expression of transporters at the BBB in healthy and pathological conditions
[2] Establishment of relationship between the level of the expression of the specific transporter at the BBB and Kp,uu,brain
[3] Generation of species specific BBB models and estimation of Kp,uu,brain
[4] Wider implementation of translational brain imaging technologies (e.g. PET) in CNS drug development programs
[5] Better understanding of the regional differences in Kp,uu,brain and how that impacts the translation of data from preclinical systems
[6] Expansion on understanding of CSF exposure
[7] Development of mathematical models (e.g. PBPK(PD), scaling of PK/PD models)
[8] Other, please specify

46. What in your opinion are the key developments needed in the Kp,uu,brain concept in the coming 15 years? Select all that apply.

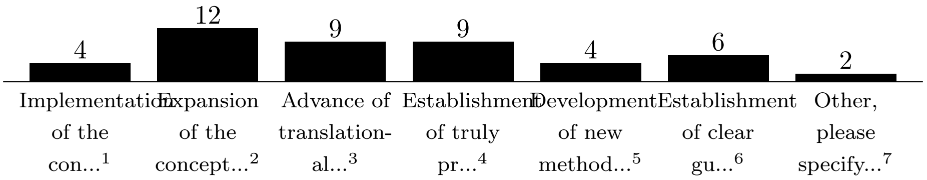

[1] Implementation of the concept into BBB cell culture models
[2] Expansion of the concept towards large molecules including therapeutic antibodies
[3] Advance of translational PBPK models
[4] Establishment of truly predictable QSAR models
[5] Development of new methods for assessment of Kp,uu,brain
[6] Establishment of clear guidance on Kp,uu,brain assessment from regulatory agencies
[7] Other, please specify

OTHER, PLEASE SPECIFY: Generation of additional Kp,uu data in higher species (e.g. monkeys, pigs) and human Kp,uu data. Better understanding of other efflux transporters besides P-gp and BCRP, and how these translate to humans, Impact of metabolism in the CNS. Regional differences. Novel uptake mechanisms

47. In my opinion the question related to Kp,uu,brain that needs the most attention is. Please add free text.

- Some outliers that can not be predicted well with current knowledge. Also for question # 43, please add the following comments: implementation of the concept has led to the development and PBPK-based scaling of in vitro reagents (MDR, BCRP) to predict kpuu from in vitro and even in silico values - thereby decreasing animal use.
- 1. Species differences in efflux transporters (expression and function). 2. Increased understanding of efflux mechanisms and involved transporters in situations where low Kp,uu can not be readily predicted (e.g. compounds with low Kp,uu despite having high passive permeability with no efflux from P-gp or BCRP expressed cell systems)
- The lack of human data is the major hurdle to better understand the relevance of species differences in transporter expression on Kpuu. Exchange between people involved, e.g. from academia and industry about best practices, case examples and pitfalls.
- All translational aspects of Kpuu from animals to humans. Expansion of the concept to other therapeutic modalities eg. human monoclonal antibodies and siRNA
- Is it reflecting the true in vivo situation? how to get the data less labor intensive?

- None
- 46 :-)
- Translation to human
- Better understanding of where the error or noise comes from in the assessment of Kpuu. Currently there are multiple measurements that factor into the Kpuu determination - plasma concentration, brain concentration, plasma binding, brain binding. Each has an associated error and underlying assumptions. Thus, there is some need to be flexible in the interpretation of a Kpuu of a value that is not ”1”. Better resolution of the Kpuu determination would be of value.
- Acceptance criteria as to what constitutes a robust Kpuu measurement for downstream quantitative applications; Assessment of specific brain site of action concentrations
- How to deliver non-small molecules to the brain
- n/a
- Kp,uu,brain of specific target cell sub-type populations; Approaches to elucidate mechanistically Kp,uu values that are or appear contradictive to the project context
- Education of clinical development colleagues, clinicians, and regulators
